# Supramolecular Annihilator with DPA Parallelly Arranged by Multiple Hydrogen-Bonding Interactions for Enhanced Triplet–Triplet Annihilation Upconversion

**DOI:** 10.3390/molecules29102203

**Published:** 2024-05-08

**Authors:** Qiuhui He, Lingling Wei, Cheng He, Cheng Yang, Wanhua Wu

**Affiliations:** Key Laboratory of Green Chemistry & Technology, College of Chemistry, Sichuan University, 29 Wangjiang Road, Chengdu 610064, China; heqiuhui77@163.com (Q.H.); weilingling375@163.com (L.W.); 15528411793@163.com (C.H.)

**Keywords:** upconversion, triplet–triplet annihilation, supramolecular annihilators, hydrogen-bonding

## Abstract

The triplet annihilator is a critical component for triplet–triplet annihilation upconversion (TTA-UC); both the photophysical properties of the annihilator and the intermolecular orientation have pivotal effects on the overall efficiency of TTA-UC. Herein, we synthesized two supramolecular annihilators **A-1** and **A-2** by grafting 9,10-diphenylanthracene (DPA) fragments, which have been widely used as triplet annihilators for TTA-UC, on a macrocyclic host—pillar[5]arenes. In **A-1**, the orientation of the two DPA units was random, while, in **A-2**, the two DPA units were pushed to a parallel arrangement by intramolecular hydrogen-bonding interactions. The two compounds showed very similar photophysical properties and host–guest binding affinities toward electron-deficient guests, but showed totally different TTA-UC emissions. The UC quantum yield of **A-2** could be optimized to 13.7% when an alkyl ammonia chain-attaching sensitizer **S-2** was used, while, for **A-1**, only 5.1% was achieved. Destroying the hydrogen-bonding interactions by adding MeOH to **A-2** significantly decreased the UC emissions, demonstrating that the parallel orientations of the two DPA units contributed greatly to the TTA-UC emissions. These results should be beneficial for annihilator designs and provide a new promising strategy for enhancing TTA-UC emissions.

## 1. Introduction

Upconversion (UC) is a phenomenon by which low-energy light is converted into high-energy light. There are several mechanisms to achieve UC emissions, such as multistep excitation of lanthanides ions [1], two-photon absorption [2], and second-harmonic generation [3], among which UC based on triplet–triplet annihilation (TTA) [4,5,6,7] is attracting continuous attention due to its unique advantages of tunable excitation wavelengths and high quantum efficiency. Thus, this has wide application potentials, ranging from photocatalysis [8,9], bio-imaging and bio-sensing [10,11,12,13], and drug release [14,15], to 3D printing [16,17]. The triplet sensitizer and annihilator are the essential components for TTA-UC, and a diagram illustrating the mechanism of TTA-UC is shown in Figure S1, in which the triplet–triplet energy transfer (TTET) process from the sensitizer to the annihilator and triplet–triplet annihilation (TTA) between the triplet annihilators are two pivotal processes for TTA-UC, both of which follow a Dexter mechanism. To facilitate the TTET process, photophysical properties of sensitizers [18,19,20] are optimized by either enhancing the visible-light absorption ability and/or extending the lifetime of their triplet states, and abundant sensitizers ranging from transition-metal complexes to metallic porphyrins and surface-engineered semiconductor nanocrystals were developed for TTA-UC [21,22,23,24]. Moreover, strategies for supramolecular pre-assembling of the sensitizers and annihilators in close proximity were also developed to facilitate the TTET process by allowing the TTET event to proceed in an intramolecular fashion instead of an intermolecular one [25,26,27,28,29]. On the other hand, for the TTA process, in which only annihilators participate, there has been far less investigation. As a matter of fact, there are three factors that jointly determine the TTA efficiency: (1) the competition between annihilation and decay for the fate of annihilators’ triplet states, (2) the collision probability of the two annihilators’ triplet states, and (3) the spin statistical factor (*η*), which gives the probability that a bright singlet state is formed from a pair of annihilating triplet states. Chemical modification of the annihilators is necessary to enhance the TTA efficiencies. Different types of dimeric annihilators based on DPA [30,31,32,33] and tetracene [34,35], as well as some DPA-based oligomers [36] and dendrimers [37], were synthesized; the triplet lifetime of the annihilator was found to significantly influence the UC emissions [30]. Also, achieving TTA in an intramolecular fashion instead of intermolecularly facilitated the TTA process. Significantly, theoretical simulations by Clark et al. showed that parallel chromophores gave higher *η* than perpendicular chromophores [38]. However, the currently developed dimers/oligomer of the annihilators have never achieved parallel arrangement of the annihilator units.

Supramolecular assembly presents an excellent strategy to precisely arrange and control the orientation of molecules [39,40,41,42,43,44,45]. Previously, we have demonstrated that chemically tuning the structures of sensitizers, as well as the host–guest complexation, could significantly facilitate the TTET process [46,47], and, by carefully regulating the aggregation state of the annihilator, the TTA process was selectively manipulated without disturbing the TTET process [48,49]. Moreover, by self-assembling the annihilators on the edge of an inorganic clay nanosheet through electrostatic attraction, a UC quantum yield (*Φ_UC_*) record in the solid state was achieved [50]. These results demonstrated that, in addition to the photophysical properties of the UC components, the orientation of molecules also had an important effect on the TTA-UC efficiencies. Thus, herein, we designed two novel supramolecular annihilators, **A-1** and **A-2**, to confirm the important effect of the molecular orientation on the TTA process. It was found that, along with the facilitated TTET and TTA process, due to the close proximity of the sensitizer and annihilator, the parallel orientation of the DPA units in **A-2** greatly enhanced the TTA process, with UC quantum yield enhanced from 5.1% for **A-1** to 13.7% for **A-2**.

## 2. Results and Discussion

### 2.1. Synthesis

**Scheme 1:**
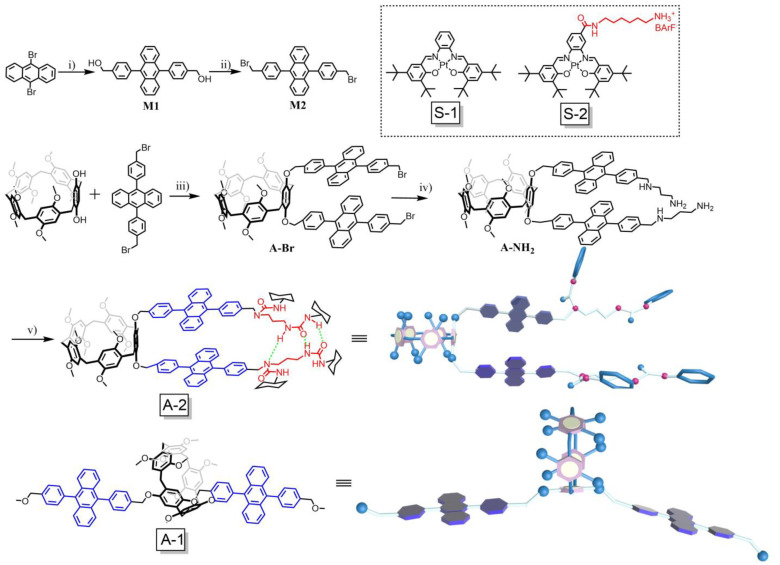
The synthetic route of the annihilator **A-2** (the green dashed line indicates hydrogen-bonding interactions) and the chemical structures of the annihilator **A-1** and the sensitizers **S-1** and **S-2**; Schematic diagrams of **A-1** and **A-2** prepared by 3DMAX software (version 2024.1) are also shown, where the big blue ball represents methoxy, the small blue ball represents oxygen atoms, and the red ball represents nitrogen atoms. Reagents and conditions: (i) (4-(hydroxymethyl) phenyl) boronic acid, [Pd(PPh_3_)_4_], K_2_CO_3_, Toluene/EtOH/H_2_O, reflux; (ii) CBr_4_, PPh_3_, CH_3_CN, RT; (iii) NaH, THF; (iv) 1,3-diaminopropane, THF; (v) cyclohexyl isocyanate, CH_3_CN.

The syntheses of **A-1** and **A-2** are outlined in Scheme 1, while the synthesis of the sensitizers is shown in Scheme S1. The intermediate **M2** was prepared by a palladium-catalyzed Suzuki cross-coupling of 9,10-dibromoanthracene with (4-(hydroxymethyl) phenyl) boronic acid, followed by a bromination of **M1** with CBr_4_. The intermediate **P[5]OH** was prepared by following a reported procedure, and the detailed description of the synthesis is displayed in the supporting information. **A-Br** was then prepared by a nucleophilic substitution reaction of **P[5]OH** and **M2.** Methanolysis of **A-Br** afforded the target product **A-1** in good yield of 85.3% over 4 steps. Reaction of **A-Br** with 1,3-diaminopropane, followed by ammonolysis of cyclohexyl isocyanate with **A-NH_2_**, gave the other product **A-2** in satisfying yield of 67.7% over 5 steps (Scheme 1). The chemical structures of **A-1**, and **A-2**, and all the intermediates were explicitly verified by standard methods of ^1^H-NMR, ^13^C-NMR, and high-resolution MS (Figures S12–S17).

### 2.2. Photophysical Properties of the Annihilators and Sensitizers

**Figure 1:**
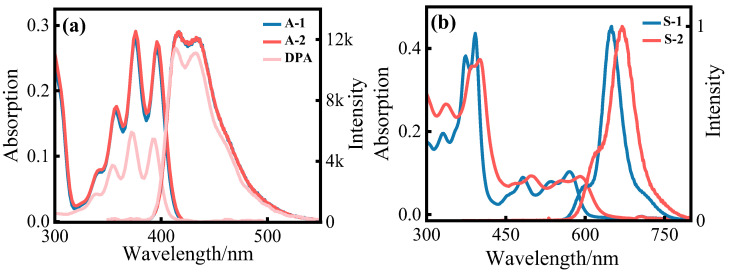
(**a**) The absorption spectra and emission spectra of the annihilators DPA, **A-1**, and **A-2**; 10 μM in toluene, 25 °C. (**b**) The absorption spectra and normalized emission spectra of the annihilators **S-1** and **S-2**; λ_ex_ = 535 nm, 10 μM in toluene at 25 °C.

The absorption and emission spectra of the supramolecular annihilators **A-1** and **A-2** are shown in Figure 1a. **A-1** and **A-2** showed very similar absorptions to that of DPA. The molar extinction coefficient (*ε*) of **A-1** and **A-2** was about twice that of DPA, proportional to the number of DPA units tethering on the pillar[5]arenes, demonstrating that there was no significant interaction between the DPA units in the ground state (Figure 1a). Moreover, **A-1** and **A-2** showed almost the same emissions, in both wavelength and intensities, and the lifetimes of the fluorescence were also comparable, which were 4.80 ns and 4.73 ns, respectively (Figure S21). The fluorescence quantum yields of **A-1** and **A-2** decreased a little bit when compared with that of DPA [51], for which the locally concentrated DPA units in **A-1** and **A-2** was most probably responsible. However, the quantum yields of **A-1** and **A-2** were comparable with each other, e.g., 65.3% for **A-1** and 62.9% for **A-2** (Table 1) in toluene, demonstrating that, despite the intramolecular hydrogen-bonding interaction between the carbamido groups in **A-2**, the excited states’ properties of the DPA units were not significantly changed and no π–π stacking between the anthracene core of DPA units was induced, which is highly beneficial for application as the annihilator in TTA-UC.

Pt (II)–salophen complexes were employed as sensitizers for the TTA-UC. The triplet lifetimes of normal Pt (II)–salophen complexes were relatively short, e.g., for **S-1,** the lifetime was determined to be only 4.4 μs (Figure S22), which was unsatisfactory for an efficient TTET process in TTA-UC in solutions [47]. Thus, supramolecular host–guest interactions to position the sensitizer and annihilator in close proximity were highly necessary. Therefore, **S-2** was prepared with an alkyl ammonia chain attached on the salophen ligand as the supramolecular binding site for the supramolecular annihilators **A-1** and **A-2**. The lifetime of **S-2** was determined to be 2.48 μs, which was comparable with **S-1.** Despite the non-extended triplet state lifetime, the host–guest complexation between **S-2** and **A-1/A-2** significantly facilitated the TTET process, which is discussed hereafter. The absorption and emission wavelength were bathochromically shifted in **S-2** due to the introduction of an amido bond. The maximum phosphorescence wavelength of **S-2** red-shifted to 670 nm (1.85 eV, Figure 1b). But, fortunately, the triplet energy level of **S-2** was still higher than that of DPA units (1.77 eV) after derivatization, which is essential for the triplet–triplet energy transfer process in TTA-UC. The photophysical parameters of the annihilators and sensitizers are summarized in Table 1.

**Figure 2:**
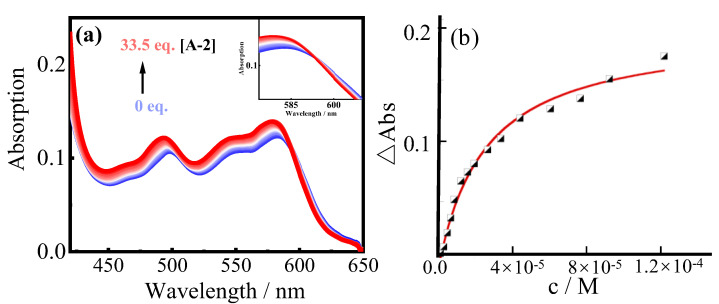
(**a**) UV–vis absorption spectra of the complexes **S-2**⊂**A-2** with different molar ratios in CHCl_3_ at 25 °C, [**S-2**] = 2.0 × 10^−5^ M, [**A-2**] = 0–0.67 mM (inset: zoomed-in view of the isoabsorptive point). (**b**) The non-linear curve-fitting (UV–vis titrations) for the complexation of **A-2** with **S-2** (2.0 × 10^−5^ M) in CHCl_3_ at 25°C; the association constant (*Ka*) was 3.6 (±0.3) × 10^4^ M^−1^. The function used to fit the data: y = 0.5 (△ε^2^x+ [G_0_] + 1/*Ka*) − (△ε^2^(x + [G_0_] + 1/*Ka*)^2^ − 4△ε[G_0_]x)^0.5^, △*ε*: difference in the molar extinction coefficient of the host before and after coordination; y: △Abs; x: concentration of the host; [G_0_]: concentration of the guest; *Ka*: the association constant.

The binding behavior of the supramolecular annihilators **A-1** and **A-2** toward **S-2** was investigated. By gradually adding **A-2** to the solutions of **S-2,** the absorption of **S-2** was blue-shifted and continuously increased (Figure 2a); an apparent isoabsorptive point at 590 nm was observed (Figure 2a inset), demonstrating a significant host–guest interaction between **A-2** and **S-2**, and the same was true for **A-1** (Figure S23a). More interestingly, the host–guest interaction significantly recovered the phosphorescence of **S-2** (Figure S24), for which the inhabitation of the photoinduced electron transfer (PET) from the Pt (II)–salophen complex to the amino cation should be most probably responsible. The binding constants for **S-2**/**A-2** were determined to be 3.6 × 10^4^ M^−1^ (Figure 2b), while, for **S-2**/**A-1**, it was 5.4 × 10^4^ M^−1^ (Figure S23b). We ascribe the slightly smaller binding affinity of **A-2** toward **S-2** than that of **A-1** to the unfavorable conformation of pillar[5]arenes units when intramolecular hydrogen-bonding in **A-2** limited the orientation of one benzene ring of the pillar[5]arene.

### 2.3. Applications of ***A-1*** and ***A-2*** as Annihilators for TTA-UC

**Figure 3:**
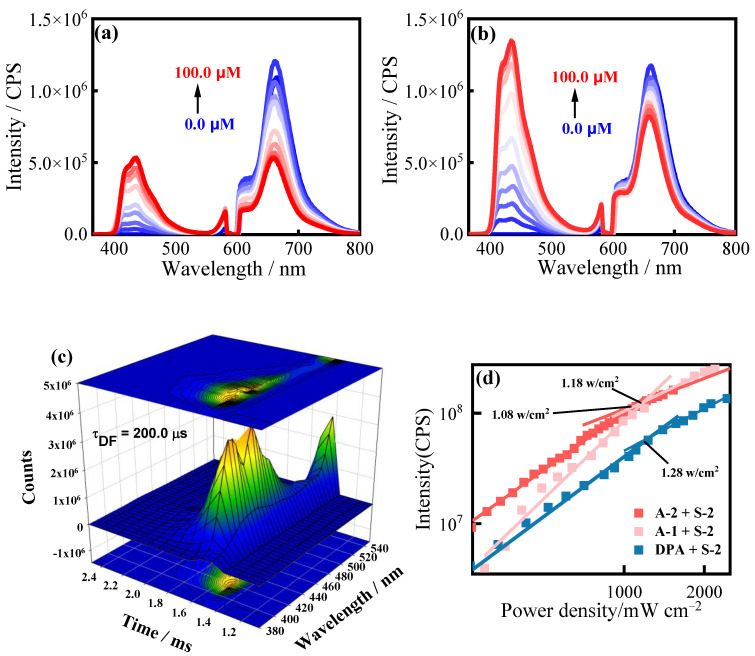
Emission spectra of the UC systems with **S-2** as the sensitizer and different concentrations of **A-1** (**a**) and **A-2** (**b**) as the annihilator. [**S-2**] = 5 μM, [annihilators] = 0.0 μM to 100.0 μM in de-aerated toluene, excited with a 589 nm laser (5.2 mW); (**c**) Time-resolved emission spectra (TRES) of the upconverted fluorescence of **A-2** using **S-2** as the photosensitizer. [**S-2**] = 5 μM, [**A-2**] = 25 μM. The change in color from blue to green to yellow represents the change in the number of photons at different wavelengths over time. (**d**) The UC intensity of UC systems **S-2**/**A-1**, **S-2**/**A-2**, and **S-2**/DPA, plotted as a function of incident light power. [**S-2**] = 5 μM, [annihilators] = 25 μM in toluene.

Upconverted emissions of the annihilators **A-1** and **A-2** were investigated *via* irradiation of the sensitizer **S-2** with a 589 nm diode-pump solid-state (DPPS) laser. As shown in Figure 3a,b, **S-2** alone showed red phosphorescence at 600–800 nm, while gradually adding **A-1** or **A-2** from 0.0 µM to 100.0 µM to the **S-2** solution, the phosphorescence of **S-2** gradually decreased, while a new emission at 400–500 nm appeared. The emissions at 400–500 nm were essentially linearly dependent on the acceptors’ concentrations at the first stage, while, after adding more than 100.0 µM of **A-2**, the emission at 400–500 nm no longer increased. The vibration structures of emission spectra were quite similar to the prompt fluorescences of **A-1** and **A-2**, and irradiating **A-1** and **A-2** alone with the 589 nm laser did not result in such emissions. Thus, the emission at 400–500 nm was ascribed to the sensitized upconverted emission of **A-1**/**A-2** by **S-2**. Time-resolved emission spectra showed that the lifetimes of the emissions at 400~500 nm were 247 μs and 200 μs for **A-1** and **A-2**, respectively (Figures S25 and S26 and Figure 3c), which were overwhelmingly longer than the prompt emissions (4.80 ns for **A-1**; 4.73 ns for **A-2**), further proving the sensitized mechanisms. The upconversion intensity of both **A-1** and **A-2** were significantly increased by increasing the power density (Figure 3d and Figure S27); the double-logarithmic plots of the integral area of UC emission vs. the power density first followed a quadratic process and then switched to a linear process. The TTA-UC threshold excitation intensity (*I_th_*) values of the **A-1/S-2** and **A-2/S-2** systems were determined to be 1.18 W/cm^2^ and 1.08 W/cm^2^, respectively, both of which were lower than that of the DPA/**S-2** system (Figure 3d, Figures S28 and S29), demonstrating that the TTA-UC processes of **A-1** and **A-2** are more efficient than that of DPA.

**Figure 4:**
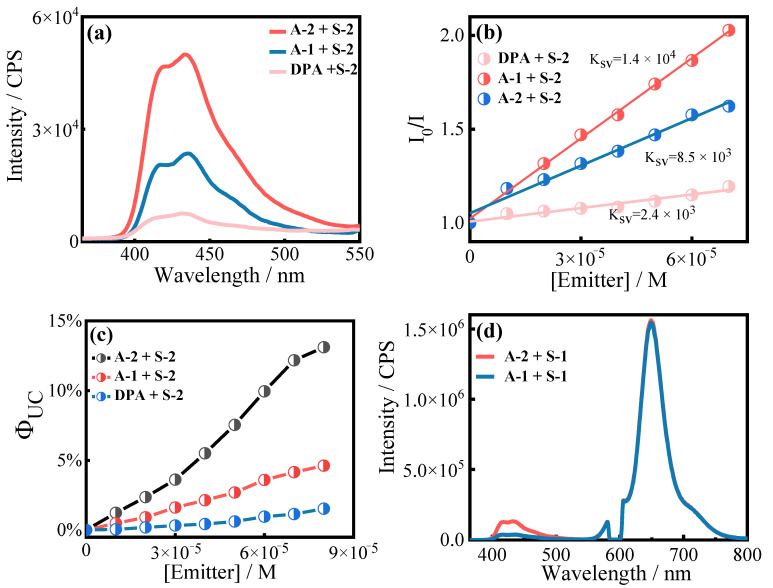
(**a**) UC emission spectra of **A-1**, **A-2**, and **DPA** with **S-2** as the sensitizer in de-aerated toluene, [**S-2**] = 5 μM, [**A-1**] = 25 μM, [**A-2**] = 25 μM, [DPA] = 50 μM; (**b**) Stern–Volmer Plots of I_0_/I versus the concentration of annihilators. [**S-2**] = 5 μM, [annihilator] = 0–70 μM in de-aerated toluene; (**c**) TTA-UC quantum yields (*Φ_UC_*) as a function of the concentration of annihilators **A-1**, **A-2**, and DPA in de-aerated toluene (with a theoretical maximum efficiency of 100%); (**d**) Emission spectra of **A-1** and **A-2** with **S-1** as the sensitizer in de-aerated toluene, [**S-1**] = 5 μM, [**A-1**, **A-2**] = 25 μM.

Indeed, the UC emissions of both **A-1** and **A-2** when **S-2** was the sensitizer were much higher than that of DPA (Figure 4a), which was as expected, as the TTET process should be significantly facilitated by the host–guest interactions in **A-1** and **A-2** [46]. To prove this, the TTET processes were investigated based on the variation in phosphorescence intensity of **S-2** with changes in the concentration of the annihilators. Increasing the concentration of **A-1**, **A-2**, and DPA led to a progressive decrease in phosphorescence intensity, and the Stern–Volmer analysis of the phosphorescence quenching data showed an approximately linear relationship (Figure 4b). The *K_SV_* values, when **A-1** and **A-2** were the acceptors, were indeed much higher than that of DPA, demonstrating that the TTET processes were facilitated by the host–guest interactions, which led to higher UC emissions.

Interestingly, the UC emission of **A-2** was much higher than that of **A-1**, e.g., at the concentration of 25 μM, the UC intensity of **A-2** was almost doubled when compared with that of **A-1** (Figure 4a). We investigated the TTA-UC quantum yield (*Φ_UC_*) at different concentrations of annihilators ranging from 10 µM to 80 μM in toluene and found that the *Φ_UC_* significantly increased upon adding different concentrations of annihilators. *Φ_UC_* reached to a plateau at the concentration of 80 μM for both **A-1** and **A-2** (Figure 4c). It was interesting to find that, at any concentration, the *Φ_UC_* of **A-2** was constantly higher than that of **A-1**, and the maximum *Φ_UC_* was determined to be 13.7% for **A-2**, which was more than two-fold higher than that of **A-1** (5.1%). These results were a little bit unexpected, as, for both **A-1** and **A-2,** host–guest interaction with the sensitizer **S-2** existed and binding constants were comparable. **A-1** showed even higher K_SV_ than **A-2** (K_SV_ = 1.4 × 10^4^ M^−1^ s^−1^ for **A-1** and 8.5 × 10^3^ M^−1^ s^−1^ for **A-2**) due to the slightly larger binding constants, demonstrating that the TTET efficiency is higher for **A-1** than for **A-2**. But, for TTA-UC emissions, **A-1** was much lower. We chose the other complex **S-1** with no supramolecular binding site as the sensitizer and found that, by adding the same concentrations of **A-1** and **A-2**, the residual phosphorescence of **S-2** was almost the same, demonstrating a comparable TTET process. However, the UC emission of **A-2** was also much higher than **A-1** (Figure 4d).

**Figure 5:**
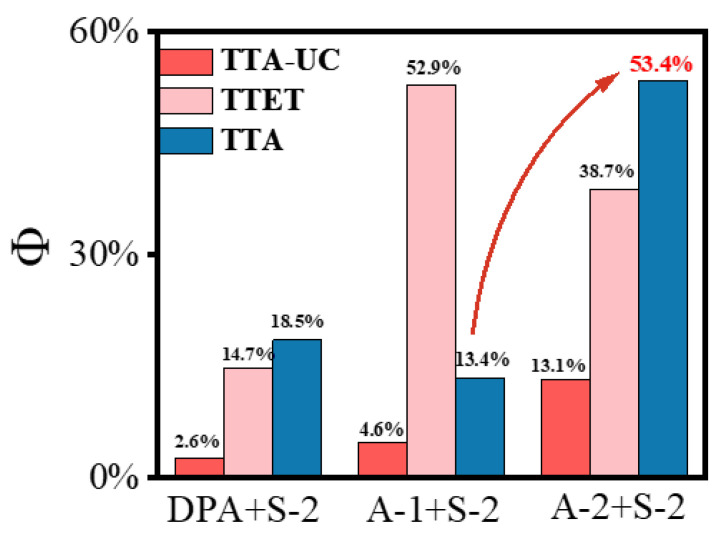
Histogram of *Φ_UC_*, *Φ_TTET_*, and *Φ_TTA_* of the UC systems with **S-2** as the sensitizer and **A-1**, **A-2**, and DPA as the annihilator. [**S-2**] = 5 μM, [**A-1**] = 80 μM, [**A-2**] = 80 μM, and [**DPA**] = 160 μM in toluene, the red arrow was used to show the increasement of *Φ_TTA_*.

The efficiency of TTA-UC fluorescence is an accumulative result of the efficiencies of all of the processes involved in the upconversion, and the *Φ_UC_* was generally written as *Φ_UC_* = *Φ_ISC_* × *Φ_TTET_* × *Φ_TTA_* × *Φ_F_*, where *Φ_ISC_*, *Φ_TTET_*, *Φ_TTA_*, and *Φ_F_* are the quantum yields of intersystem crossing of the sensitizer, triplet energy transfer from the sensitizer to the annihilator, the acceptor triplet annihilation, and acceptor fluorescence, respectively. Considering the even lower TTET process for **A-2** than **A-1** when **S-2** was the sensitizer and the comparable fluorescence quantum yield of **A-1** and **A-2** (according to the above equation), the much higher Φ_UC_ of **A-2** was, therefore, ascribed to the significantly improved Φ_TTA_. We carefully quantified the efficiencies of the main processes (TTET and TTA processes) involved in TTA-UC, as shown in Figure 5, it was found that, when **S-2** was the sensitizer, the TTET efficiency of **A-1** (80 μM) was determined to be 52.9%, which was higher than that of **A-2** (38.7%). However, for the TTA efficiencies, only 13.4% was observed for **A-1**, but 53.4% was observed for **A-2**; the TTA process in **A-2** was much more efficient than that of **A-1.** Similar results were observed when **S-1** was the sensitizer (Figures S30 and S34): the TTA efficiency of **A-2** was also three-fold higher than that of **A-1** (16.3% for **A-1** and 51.4% for **A-2**). As mentioned earlier, for the TTA process, the following aspects should jointly contribute to the efficiency: the competition between annihilation and decay for the fate of triplet states, the collision probability of the two triplet states of the annihilator, and the spin statistical factor (*η)* [38]. Herein, the first influencing factor should be the same, as same fluorophore DPA was used in both **A-1** and **A-2**. However, the orientations of the two DPA units in **A-1** and **A-2** were different: the orientation of the two DPA units in **A-1** was random, while, in **A-2**, the two DPA units were pushed to a parallel arrangement by intramolecular hydrogen-bonding interactions. The collision probability of the triplet states of DPA units in **A-2** should be higher due to the close proximity of the two DPA units. Also, the probability of forming a singlet state from a pair of annihilating triplet states (the spin statistical factor *η*) should also be higher, according to a recent theoretical simulation that showed that parallel chromophores gave higher *η* than perpendicular chromophores [38]. Thus, for the first time, we experimentally confirmed that the parallel orientation of the two annihilators significantly enhanced the TTA efficiencies.

**Figure 6:**
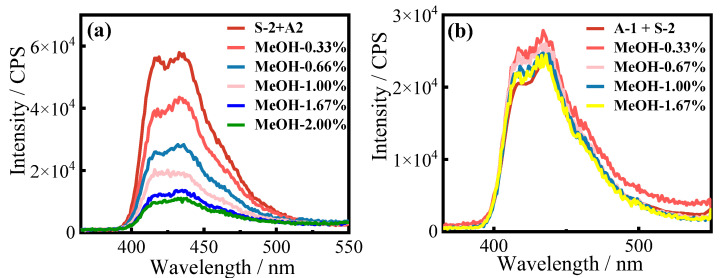
(**a**) UC emission spectra of UC systems **A-2**/**S-2** (**a**) and **A-1**/**S-2** (**b**) in de-aerated toluene with different concentrations of MeOH. [**S-2**] = 5 μM, [**A-1**, **A-2**] = 25 μM.

To further confirm the pivotal effect of the parallel arrangement of two DPA units by hydrogen-bonding interaction on the TTA efficiencies, the solvent for TTA-UC was changed to MeOH, which is well-known to destroy the hydrogen-bonding interactions. It was found that the UC emissions of both **A-1** and **A-2** in MeOH were significantly decreased, but the intensities for the two compounds were comparable (Figure S35). More interestingly, we gradually added trace methanol, from 0.33% to 2.00%, to the toluene solution of **A-2/S-2**, i.e., concentrations for which we thought the polarity of the solvent would not change much. The UC emission of **A-2** was significantly decreased (Figure 6a), while adding the same amount MeOH to the **A-1**/**S-2** system did not result in such change—the UC emission basically remained unchanged (Figure 6b). These results demonstrated again that the more efficient TTA-UC for **A-2** was due to parallel arrangement of DPA units by hydrogen-bonding interactions.

## 3. Materials and Methods

All chemicals were obtained from commercial suppliers and used as received. ^1^HNMR and ^13^C NMR spectra in CDCl_3_, DMSO were obtained using a Brucker DRX-400 spectrometer (Billerica, MA, USA). HRMS was determined by MALDI-TOF–MS. UV–vis spectra and fluorescence spectra were recorded using a Duetta spectrometer (Horiba, Kyoto, Japan). Upconversion measurements were performed using a diode-pumped solid-state laser (589 nm or 532 nm) (Minghui Optoelectronic Technology, Xi’an, China), synchronized with a fluoromax-4 fluorescence spectrometer (Horiba, Kyoto, Japan). Fluorescence and upconversion lifetime attenuation are also performed on fluoromax-4 fluorescence spectrometers.

### 3.1. Synthesis of ***A-1*** and ***A-2***

The syntheses of intermediates **M1**, **M2**, and **P5-Q** are shown in the support information.

**A-Br**: To a mixture of compound **P5-Q** (0.27 g, 0.36 mmol) in THF (20 mL), we added NaBH_4_ (0.30 g, 7.9 mmol), which was dissolved in MeOH (5 mL), and the reaction mixture was stirred at 25 °C for 20 min. At the end of the reaction, CH_2_Cl_2_ (150 mL) was added to the reaction mixture and then poured into the dilute hydrochloric acid aqueous solution. Then, the organic phase was dried over Na_2_SO_4_ and the solvent was removed under reduced pressure to afford the intermediate as a white solid. Then, a mixture of the white intermediate (0.16 g, 0.23 mmol), **M2** (0.35 g, 0.681 mol) in THF (50 mL), was stirred. After bubbling N_2_ through the mixture three times, NaH (0.16 g, 0.69 mmol) was added, and the mixture was stirred and refluxed at 70 °C for 10 h. The solution was concentrated under reduced pressure to afford the crude product, which was further purified via silica gel chromatography (PE:CH_2_Cl_2_ = 2:1) to afford compound **A-Br** as a light-yellow solid (0.06 g, 16.4%). ^1^H NMR (400 MHz, CDCl_3_):δ 7.71 (d, *J* = 11.9 Hz, 17H), 7.52 (s, 8H), 7.39 (s, 9H), 7.13 (s, 2H), 6.92 (s, 2H), 6.83 (s, 2H), 6.79 (s, 4H), 5.17 (d, *J* = 17.9 Hz, 5H), 4.73 (s, 4H), 4.15 (d, *J* = 6.8 Hz, 2H), 3.93 (d, *J* = 12.9 Hz, 2H), 3.80 (d, *J* = 5.0 Hz, 6H), 3.72 (s, 6H), 3.64 (d, *J* = 7.7 Hz, 12H), 3.56 (s, 6H). ^13^C NMR (101 MHz, CDCl_3_): δ 150.8, 150.8, 150.8, 150.4, 139.3, 138.4, 137.4, 137.1, 136.9, 136.4, 131.8, 131.5, 129.9, 129.8, 129.2, 128.8, 128.3, 128.2, 128.2, 128.1, 127.5, 126.9, 126.9, 125.3, 125.2, 70.6, 55.9, 55.8, 55.7, 55.7, 29.5. ESI-HRMS: calcd (C_99_H_84_Br_2_O_10_H]^+^), *m*/*z* = 1591.4509, found: *m*/*z* = 1591.4509; calcd ([C_99_H_84_Br_2_O_10_Na]^+^), *m*/*z* = 1613.4329, found: *m*/*z* = 1613.4338.

**A-NH_2_**: A mixture of 1,3-propane diamine (0.093 g, 1.25 mmol) and **A-Br** (0.050 g, 0.03 mmol) in THF (10 mL) was stirred at 25 °C for 12 h. The solvent and excess diamine were removed under reduced pressure; then, we dissolved the resulting oil in methanol and added potassium hydroxide (0.0035 g, 0.025 mmol) to the mixture. The ether (30 mL) was added to precipitate the inorganic salts. The mixture was filtered and concentrated under reduced pressure to afford compound **A-NH_2_** as a yellow solid (0.05 g, 42.2%).^1^H NMR (400 MHz, CDCl_3_): δ 7.77 (s, 11H), 7.66–7.46 (m, 12H), 7.38 (s, 9H), 7.14 (s, 2H), 6.93 (s, 2H), 6.82 (d, *J* = 15.2 Hz, 6H), 5.18 (d, *J* = 16.1 Hz, 5H), 4.13 (d, *J* = 12.9 Hz, 2H), 4.02 (s, 2H), 3.93 (d, *J* = 13.1 Hz, 2H), 3.80 (s, 6H), 3.72 (s, 6H), 3.64 (d, *J* = 6.8 Hz, 12H), 3.56 (s, 6H), 2.90 (s, 4H), 1.91 (s, 4H), 1.79 (s, 4H), 1.44 (s, 4H), 0.99 (s, 4H). ^13^C NMR (101 MHz, DMSO-*d*_6_): δ 172.5, 150.5, 150.3, 149.9, 137.9, 131.4, 129.8, 128.4, 127.9, 126.7, 125.9, 113.6, 71.9, 61.6, 60.8, 55.8, 55.7, 55.6, 55.4, 45.9, 36.6, 34.3, 32.7, 29.4, 29.2, 27.7. MALDI-HRMS: calcd ([C_105_H_102_N_4_O_10_H]^+^), *m*/*z* = 1579.7674, found: *m*/*z* = 1579.7663; calcd ([C_105_H_102_N_4_O_10_Na]^+^), *m*/*z* = 1601.7494, found: *m*/*z* = 1601.7465; calcd ([C_105_H_102_N_4_O_10_K]^+^), *m*/*z* = 1617.7233, found: *m*/*z* = 1617.7223.

**A-1**: A mixture of NaH (1.13 mg, 0.0471 mmol) and anhydrous methanol (1.0 mL, 3.0 eq) in THF (20 mL) was stirred at 0 °C under a N_2_ atmosphere for 30 min. Then, **A-Br** (50.0 mg, 0.0314 mmol) was added and the reaction was stirred at 25 °C for 5 h and monitored by TLC; the reaction was stopped when the raw material disappeared. The solvent was removed under reduced pressure to obtain the crude product, which was further purified via silica gel chromatography (PE:CH_2_Cl_2_ = 1:5) to afford compound **A-1** as a white solid (40.0 mg, 85.3%). ^1^H NMR (400 MHz, CDCl_3_): δ 7.76 (t, *J* = 6.1 Hz, 12H), 7.63 (d, *J* = 7.9 Hz, 4H), 7.53 (dd, *J* = 13.7, 7.8 Hz, 8H), 7.42–7.35 (m, 8H), 7.13 (s, 2H), 6.92 (s, 2H), 6.81 (d, *J* = 14.9 Hz, 6H), 5.24–5.11 (m, 4H), 4.68 (s, 4H), 4.13 (d, *J* = 12.9 Hz, 2H), 3.93 (d, *J* = 12.8 Hz, 2H), 3.80 (d, *J* = 5.5 Hz, 6H), 3.72 (s, 6H), 3.64 (d, *J* = 7.1 Hz, 12H), 3.59 (s, 6H), 3.56 (s, 6H). ^13^C NMR (101 MHz, CDCl_3_): δ 150.9, 150.8, 150.8, 150.5, 138.5, 138.4, 137.6, 137.1, 136.7, 131.5, 131.4, 129.9, 128.9, 128.2, 128.1, 127.9, 127.5, 127.1, 126.8, 125.1, 125.1, 115.6, 114.2, 77.3, 77.0, 76.7, 74.8, 70.7, 58.6, 55.9, 55.8, 30.0, 29.7, 22.7. ESI-HRMS: calcd (C_101_H_90_O_12_]^+^), *m*/*z* = 1494.6432, found: *m*/*z* = 1494.6473;calcd ([C_101_H_90_O_12_NH_4_]^+^), *m*/*z* = 1512.6771, found: *m*/*z* = 1512.6701.

**A-2**: A mixture of **A-NH_2_** (50 mg, 0.032 mmol) and cyclohexyl isocyanate (8.17 μL, 0.064 mmol) in acetonitrile (20 mL) was stirred at 25 °C for 4 h. The reaction was stopped when the raw material disappeared; then, the solvent was removed under reduced pressure to obtain the crude product, which was further purified via silica gel chromatography (CH_2_Cl_2_: MeOH = 15:1) to afford compound **A-2** as a light-yellow solid (45.0 mg, 67.7%). ^1^H NMR (400 MHz, CDCl_3_): δ 7.76 (t, *J* = 7.3 Hz, 8H), 7.68 (d, *J* = 8.8 Hz, 4H), 7.55 (d, *J* = 8.8 Hz, 12H), 7.42–7.36 (m, 8H), 7.13 (s, 2H), 6.92 (s, 2H), 6.81 (d, *J* = 15.2 Hz, 6H), 5.30 (s, 2H), 5.23–5.12 (m, 5H), 4.58 (s, 4H), 4.48 (d, *J* = 7.6 Hz, 2H), 4.35 (t, *J* = 6.5 Hz, 2H), 4.11 (s, 2H), 3.92 (d, *J* = 11.4 Hz, 2H), 3.80 (s, 6H), 3.72 (s, 6H), 3.64 (d, *J* = 7.7 Hz, 12H), 3.56 (s, 6H), 3.50 (s, 4H), 3.33 (s, 4H), 3.28 (s, 4H), 1.98–1.93 (m, 16H), 1.83 (s, 8H), 1.01–0.95 (m, 5H), 0.93–0.85 (m, 16H); ^13^C NMR (101 MHz, DMSO-*d*_6_): δ 157.1, 130.6, 130.1, 129.8, 127.9, 125.9, 79.7, 47.9, 33.8, 33.6, 31.4, 29.5, 25.8, 25.5, 24.9, 22.5, 14.4. MALDI-HRMS: calcd ([C_133_H_147_N_8_O_14_]^+^), *m*/*z* = 2080.1037, found: *m*/*z* = 2080.1008; calcd ([C_133_H_147_N_8_O_14_Na]^+^), *m*/*z* = 2102.0856, found: *m*/*z* = 2102.0846.

### 3.2. Measurement

The fluorescence quantum efficiency (*Φ_F_*) of the annihilators and sensitizers was determined by using 9,10-diphenyl anthracene (DPA) in ethanol (*Φ_F_* = 95%) and rose bengal (*Φ_F_* = 11%) as the standard, respectively. The upconversion quantum yields (*Φ_UC_*, of 100% maximum) were determined with cresol violet (*Φ_std_* = 51% in EtOH) as the standard and were calculated with the following formula, Equation (1), where *Φ_UC_* stands for the upconversion quantum yield; *A*, *I* and *η* represent the absorbance, integrated photoluminescence intensity, and refractive of the solvent, respectively; *sam* means the sample; and *std* is the standard.

(1) $$\Phi_{UC}=2\Phi_{std}\left(\frac{A_{std}}{A_{sam}}\right)\left(\frac{I_{sam}}{I_{std}}\right){\left(\frac{\eta_{sam}}{\eta_{std}}\right)}^{2}$$

The efficiencies of the triplet–triplet energy transfer (TTET) between the sensitizers and annihilators were determined by the quenching of the phosphorescence of the sensitizers and was calculated with the formula in Equation (2), where *I* and *I*_0_ stand for the integration of the emission in the presence and absence of the annihilators, respectively.

(2) $$\Phi_{TTET}=1-I/I_{0}$$

The TTA efficiencies between the annihilators were determined using Equation (3), where *Φ_F_* is the fluorescent quantum yield of the annihilator and *Φ_ISC_* is the intersystem crossing (ISC) efficiency of the sensitizer, which was approximately 1.

(3) $$\Phi_{TTA}=\frac{\Phi_{UC}}{\Phi_{TTET}\cdot \Phi_{F}\cdot \Phi_{ISC}}$$

## 4. Conclusions

In conclusion, two supramolecular annihilators, **A-1** and **A-2**, with two DPA units grafted on pillar[5]arenes were synthesized. In **A-1**, the orientation of the two DPA units was random, while, in **A-2**, the two DPA units were pushed to a parallel arrangement by intramolecular hydrogen-bonding interactions in toluene. It was found that **A-1** and **A-2** showed very similar photophysical properties, including similar fluorescence quantum yield and lifetimes. Furthermore, they also showed comparable host–guest binding affinities toward an alkyl ammonia chain-attaching sensitizer **S-2**. The supramolecular annihilators **A-1** and **A-2** showed much more efficient TTA-UC compared with the traditional DPA annihilator. The host–guest interaction between the sensitizer and annihilator significantly facilitated the TTET process. More significantly, the TTA-UC emission of **A-2** was much higher than that of **A-1**, and the UC quantum yield of **A-2** sensitized by **S-2** was determined to be 13.7%, which is more than twice that of **A-1** (5.1%). The parallel arrangement of the two DPA units by multiple hydrogen-bonding interactions, which significantly increased the TTA efficiency, was demonstrated to be responsible for the much higher TTA-UC emissions for **A-2**. This work presented a novel strategy for enhancing TTA-UC efficiency and provided design rationales for innovative supramolecular annihilator.

## Figures and Tables

**Table 1:** Photophysical parameters of the annihilators and the sensitizers ^a^.

	*λ*_abs_/nm	*ε* ^b^/10^4^ M^−1^ cm^−1^	*λ*_em_/nm	*Φ* ^c^/%	τ ^e^/ns
**A-1**	358/376/396	1.44/2.39/2.26	417, 435	65.3	4.80
**A-2**	358/376/396	1.50/2.48/2.33	417, 435	62.9	4.73
DPA	358/376/396	0.78/1.18/1.11	417, 435	95.0	5.30
**S-1**	570	1.03	650	9.94 ^d^	4400
**S-2**	585	0.90	670	2.16 ^d^	2480

^a^ 1 × 10^−5^ M in toluene. ^b^ Molar extinction coefficient. ^c^ Luminescence quantum efficiency determined by a relative method, using DPA (*Φ_F_* = 95% in EtOH) as the standard. ^d^ Rose bengal as the standard (*Φ_F_* = 11% in EtOH). ^e^ The lifetime.

## Data Availability

Data are contained within the article and Supplementary Materials.

## Supplementary Materials

The following supporting information can be downloaded at: https://www.mdpi.com/article/10.3390/molecules29102203/s1, Scheme S1: Synthesis of **S-2**, Figure S1: Schematic diagram of the conventional TTA-UC process, Figures S2–S5: 1H-NMR spectra of **P5**, **P5-Q**, **M1, M2**, Figures S6–S17: ^1^H NMR; ^13^C NMR and HRMS spectra of **A-Br, A-NH_2_, A-1, A-2**, Figure S18: ^1^H NMR spectra of **S-6A**, Figures S19 and S20: ^1^H NMR and ^13^C NMR of **S-2**, Figure S21: Fluorescence lifetime decay curves of **A-1**, **A-2**, Figure S22: Phosphorescence lifetime decay curves of **S-1** and **S-2**, Figure S23: UV–Vis absorption spectra and non-linear curve-fitting of the complexes **S-2/A-1**, Figure S24: Emission spectra of **P5, P5/S-2**, Figure S25: Delayed fluorescence spectra of **S-2/A-2**, Figure S26: Time-resolved emission spectra (TRES) and delayed fluorescence spectra of the upconverted **S-2/A-1**, Figure S27: Excitation power dependency of the upconverted **S-2/A-1** and **S-2/A-1**, Figure S28: UC emission spectra and excitation power dependency of the upconverted **S-2**/DPA, Figure S29: Delayed fluorescence spectra of the upconverted **S-2**/DPA, Figure S30: UC emission spectra and Stern–Volmer plots of the upconverted **S-1/A-1**, Figure S31: UC emission spectra and Stern–Volmer plots of the upconverted **S-1/A-2**, Figure S32:The histogram of the efficiency of the upconversion process of the upconverted **S-1/A-1**, **S-1/A-2**, Figure S33: Delayed fluorescence spectra and excitation power dependency of the upconverted **S-1/A-1**, Figure S34: Delayed fluorescence spectra and excitation power dependency of the upconverted **S-1/A-2**, Figure S35: UC emission spectra of the upconverted **S-2/A-1**, **S-2/A-2** in MeOH.
