# Supplementary material for: Supramolecular Annihilator with DPA Parallelly Arranged by Multiple Hydrogen-Bonding Interactions for Enhanced Triplet–Triplet Annihilation Upconversion
Source: Molecules. 2024 May 8;29(10):2203. doi: 10.3390/molecules29102203 (PMC11124113; doi:10.3390/molecules29102203)
Supplement: Supplementary file 1 [file molecules-29-02203-s001.zip › molecules-2967720-supplementary.pdf]

Supporting information for:

Supramolecular Annihilator with DPA Parallelly Arranged by Multiple Hydrogen-Bonding Interactions for Enhanced Triplet–Triplet Annihilation Upconversion

Qiuhui He, Lingling Wei, Cheng He, Cheng Yang * and Wanhua Wu *

Key Laboratory of Green Chemistry & Technology, College of Chemistry, Sichuan University,
29 Wangjiang Road, Chengdu 610064, China

Table of Contents

S1. Mechanism diagram of TTA-UC 2

S2. Synthesis and characterizations 2

S3. ^{1}HNMR, ^{13}CNMR and MS spectra 6

S4. Photophysical details 15

S5. Host-guest binding details 16

S6. Upconversion details 17

S1. Mechanism diagram of TTA-UC

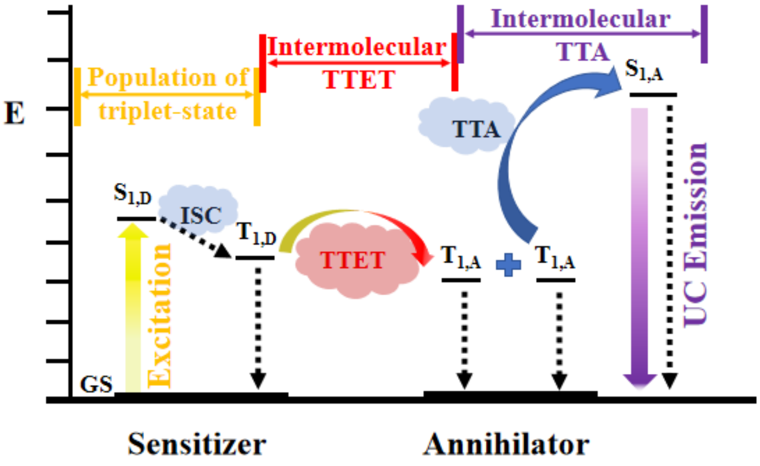

Figure S1. Schematic diagram of the conventional TTA-UC process.GS denotes the ground state, and $S_{1,D}$ and $T_{1,D}$ denote the singlet and triplet excited states of the donor (sensitizer), respectively. ISC, TTET, and TTA denote the intersystem crossover, triplet-triplet energy transfer, and triplet-triplet annihilation, respectively. $S_{1,A}$, and $T_{1,A}$ denote the singlet and triplet excited states of the acceptor, respectively. Upward solid arrows and downward dashed arrows indicate absorption leaps, emission leaps, and nonradiative leaps, respectively.

S2. Synthesis and characterizations

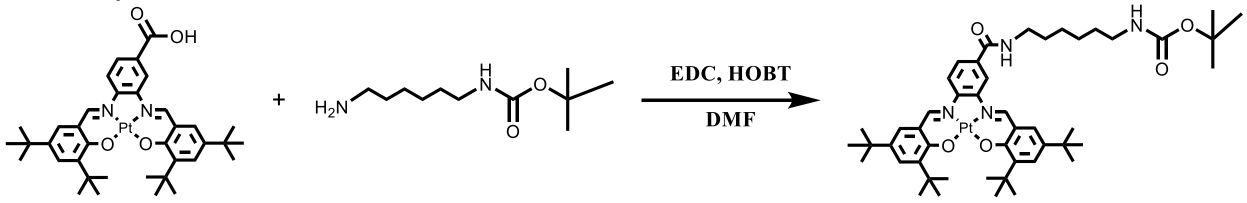

Pt-OH **B-0** **S-0**

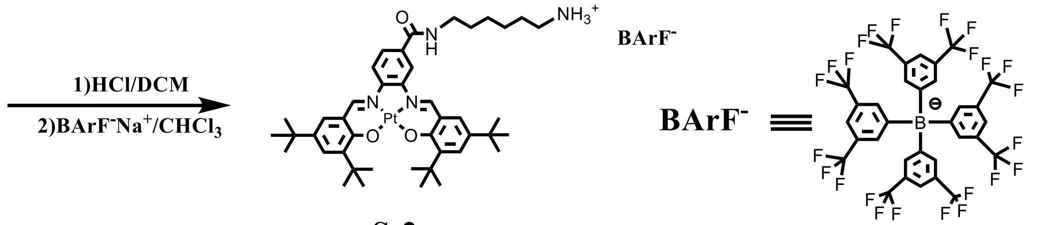

S-2

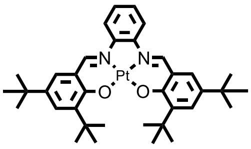

S-1

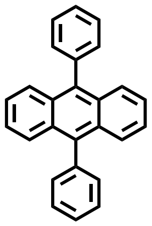

DPA

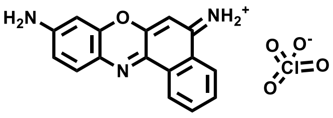

cresol purple

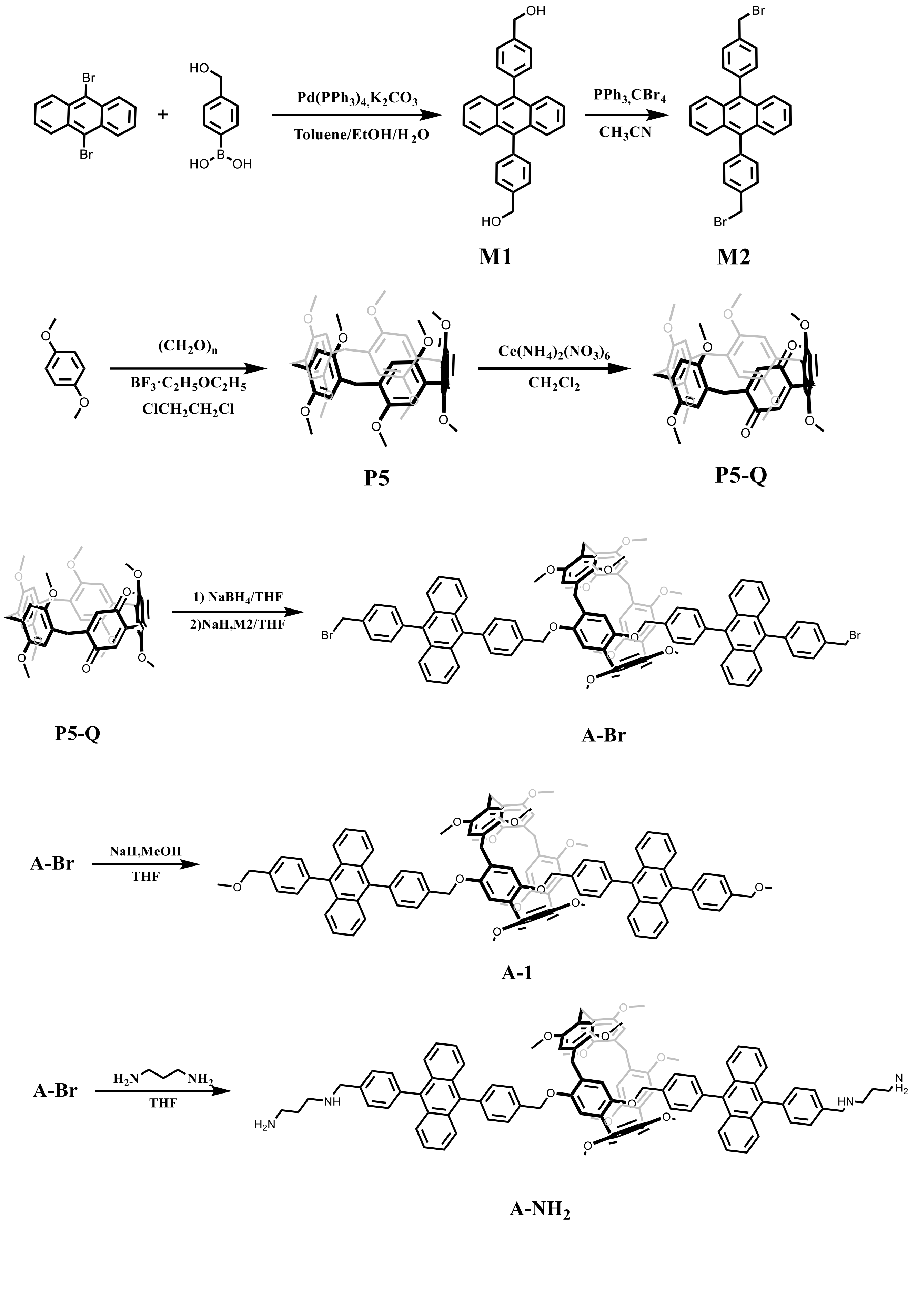

Br
Br
+
HO
HO B OH
Pd(PPh3)4,K2CO3
Toluene/EtOH/H2O
OH
HO
M1
PPh3,CBr4
CH3CN
Br
Br
M2
(CH2O)n
BF3·C2H5OC2H5
ClCH2CH2Cl
P5
Ce(NH4)2(NO3)6
CH2Cl2
P5-Q
1) NaBH4/THF
2)NaH,M2/THF
P5-Q
A-Br
A-Br
NaH,MeOH
THF
A-1
A-Br
H2N NH2
THF
NH
H2N
HN
NH2
A-NH2

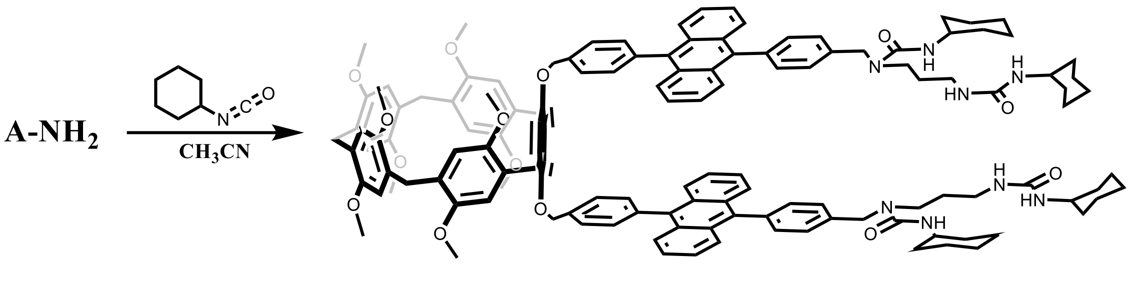

A-2

Scheme S1. Synthesis route of the acceptors **A-1**, **A-2**, the sensitizer **S-2** and the chemical structures of DPA, cresol purple.

P5: To a mixture of 1,4-dimethoxybenzene (6.00 g, 43.4 mmol), paraformaldehyde (5.21 g, 173.5 mmol) in 1,2-dichloroethane (300 mL) was added 4.33 mL BF_3OEt_2, and the reaction mixture was stirred at 25°C for 30 minutes. The reaction was stopped when the white mixture gradually turned blue-green. Washing three times with aqueous sodium bicarbonate, then washing with water to remove excess sodium bicarbonate. The organic phase was dried over Na_2SO_4 and the solvent was removed under reduced pressure to afford the crude product which was further purified by silica gel chromatography (CH_2Cl_2) to afford compound **P5** as white solid (2.0 g, 31.7%). ^{1}H NMR (400 MHz, $CDCl_3$): δ 6.79 (s, 10H), 3.81 – 3.79 (m, 10H), 3.67 (s, 30H).

P5-Q: A mixture of **P5** (1.000 g, 1.33 mmol) in dichloromethane (50 mL) was stirred, then ammonium persimmon nitrate (1.095 g, 2.00 mmol) was dissolved in water (2 mL). With the addition of ammonium persimmon nitrate solution, the color of the solution gradually changed from white to orange-red. The resulting mixture was stirred at 25°C for 30 minutes. The organic phase was washed three times with water, then the organic phase was dried over Na_2SO_4 and the solvent was removed under reduced pressure to afford the crude product which was further purified by silica gel chromatography (PE: CH_2Cl_2= 1: 6) to afford compound **P5-Q** as an orange-red solid (0.3 g, 31.3%). ^{1}H NMR (400 MHz, $CDCl_3$): δ 6.87 (s, 2H), 6.82 (d, J = 6.0 Hz, 4H), 6.69 (d, J = 3.4 Hz, 4H), 5.26 (s, 2H), 3.81 (s, 4H), 3.77 (s, 6H), 3.74 (s, 12H), 3.67 (s, 2H), 3.65 (s, 6H), 3.61 (s, 4H).

M1: An aqueous solution of K_2CO_3 (8.29 g, 6.0 mmol) were added to the solution of 9, 10-dibromo anthracene (3.36 g, 10.0 mmol) and 4-hydroxyphenylboric acid (6.84 g, 45 mmol) in the mixed solvent of toluene/ ethanol/ H_2O (60 mL/ 20 mL/ 30 mL). After bubbling N_2 through the mixture for three times, Pd $(PPh_3)_4$ (0.58 g, 0.5 mmol) was added, and the mixture was stirred and refluxed at 98°C for 24 hours. The solution was cooled to room temperature, pumped and filtered, the solid is washed with methanol, and the obtained solid was dried in vacuum for 24h to afford compound **M1** as a yellow solid (3.5 g, 89.7%). ^{1}H NMR (400 MHz, $CDCl_3$):δ 7.66 (d, J = 7.3 Hz, 8H), 7.44 (d, J = 7.9 Hz, 4H), 7.33 (d, J = 6.9 Hz, 4H), 4.84 (s, 4H);

M2: To a mixture of compound **M1** (2.00 g, 5.1 mmol), triphenylphosphine (3.66 g, 56.1 mol) in CH_3CN (150 mL) was added CBr_4 (4.24 g, 51.6 mmol) in an ice bath, and the reaction mixture was stirred for 30 minutes, then the ice bath was removed and the mixture was stirred vigorously at 25°C for 4 hours. Then water (100 mL) was added to the mixture and the crude

product was precipitated, then the precipitate was washed with MeOH and water and dried under vacuum to afford compound **M2** as a yellow solid (2.0 g, 76.6%). ^{1}H NMR (400 MHz, $CDCl_3$): δ 7.72 – 7.65 (m, 8H), 7.49 (d, J = 8.1 Hz, 4H), 7.37 (dd, *J* = 6.9, 3.2 Hz, 4H), 4.72 (s, 4H);

S-0: The mixture of compound **Pt-OH** (0.10 g, 0.128 mmol), HOBT (0.035 g, 0.256 mmol), EDC (34 μL, 0.192 mmol) were dissolved in 4.5 mL DMF. After bubbling N_2 through the mixture for three times, and activation at -20°C for 2 hours. Then N-Boc-hexanediamine (**B-0**, 55 μL, 0.256 mmol) was added and the reaction was kept in a vacuum and at 25°C under N_2 atmosphere for 6 hours. The solvent was removed under reduced pressure to afford the crude product which was further purified by silica gel chromatography (CH_2Cl_2: MeOH= 200: 1) to afford compound **S-0** as a purple solid (0.08g, 64.1%). ^{1}H NMR (400 MHz, $CDCl_3$): δ 9.10 (s, 1H), 8.94 (s, 1H), 8.76 (s, 1H), 8.05 (d, *J* = 8.7 Hz, 1H), 7.72 – 7.69 (m, 3H), 7.36 (d, *J* = 2.6 Hz, 2H), 3.53 (d, *J* = 6.2 Hz, 2H), 3.29 (d, *J* = 6.7 Hz, 2H), 1.71 (d, *J* = 6.6 Hz, 2H), 1.60 (d, *J* = 2.6 Hz, 18H), 1.56 (s, 2H), 1.44 (s, 9H), 1.38 (d, *J* = 1.7 Hz, 22H).

S-2: To a mixture of compound **S-0** (0.04 g, 0.041 mmol) in a small amount of DCM was added 1.0 mL hydrochloric ethanol solution, then the reaction mixture was stirred at 25°C for 8 hours. The solvent was removed under reduced pressure to afford the intermediate as a purple solid. Then the intermediate was placed in a round-bottomed flask, $BArF^-Na^+$ (0.078 g, 0.088 mmol) dissolved in a small amount of chloroform was added, and the reaction mixture was stirred at 25°C for 8 hours. The solution was filtered through an organic filtration membrane and the solvent was removed under reduced pressure to obtain the crude product which was further purified by silica gel chromatography (CH_2Cl_2: MeOH= 20: 1) to afford compound **S-2** as a red solid (0.064 g, 89.7%). ^{1}H NMR (400 MHz, $CDCl_3$): δ 8.70 (s, 1H), 8.20 (s, 1H), 7.69 (s, 14H), 7.50 (s, 5H), 3.32 (t, *J* = 12.1 Hz, 2H), 2.99 – 2.84 (m, 2H), 1.41 (s, 2H), 1.34 (s, 18H), 1.30 (d, *J* = 4.2 Hz, 6H), 1.26 (s, 18H). ^{13}C NMR (101 MHz, $CDCl_3$): δ 162.4, 134.7, 129.0, 128.7, 128.6, 125.9, 123.1, 117.5, 31.9, 31.1, 29.7, 29.4, 22.7.

S3. ^{1}HNMR,^{13}CNMR and MS spectra

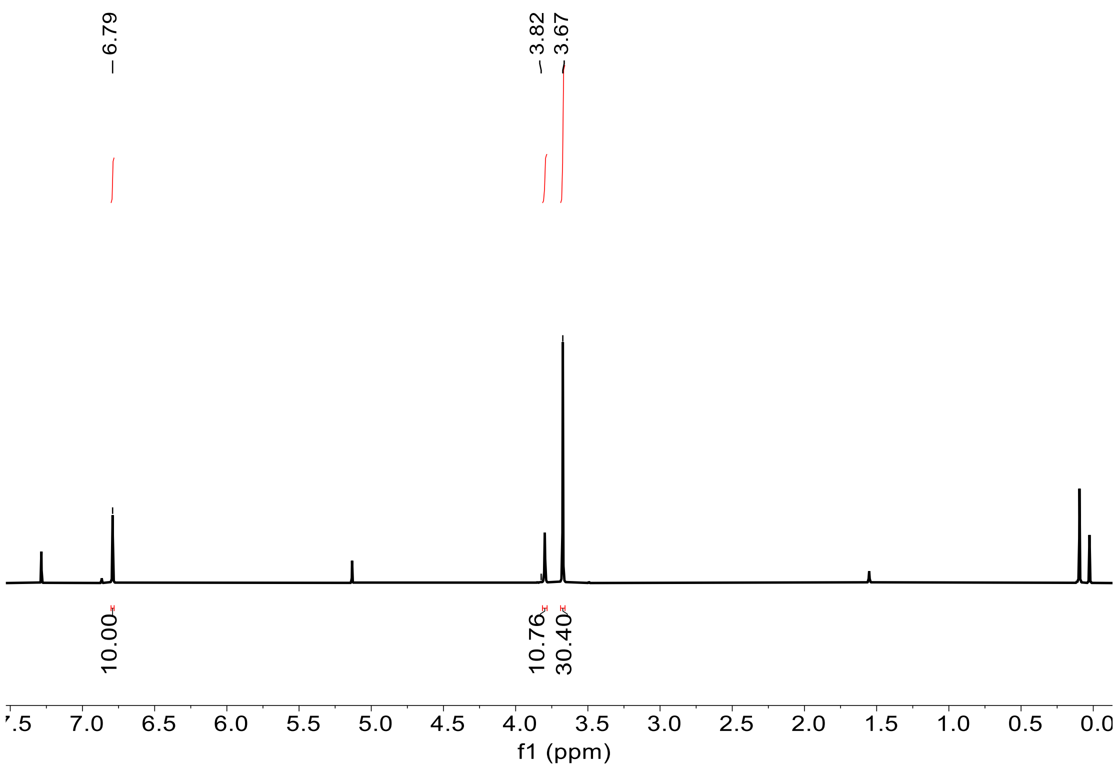

Figure S2. The ^{1}H NMR spectrum (400 MHz, $CDCl_3$, room temperature) of compound **P5**.

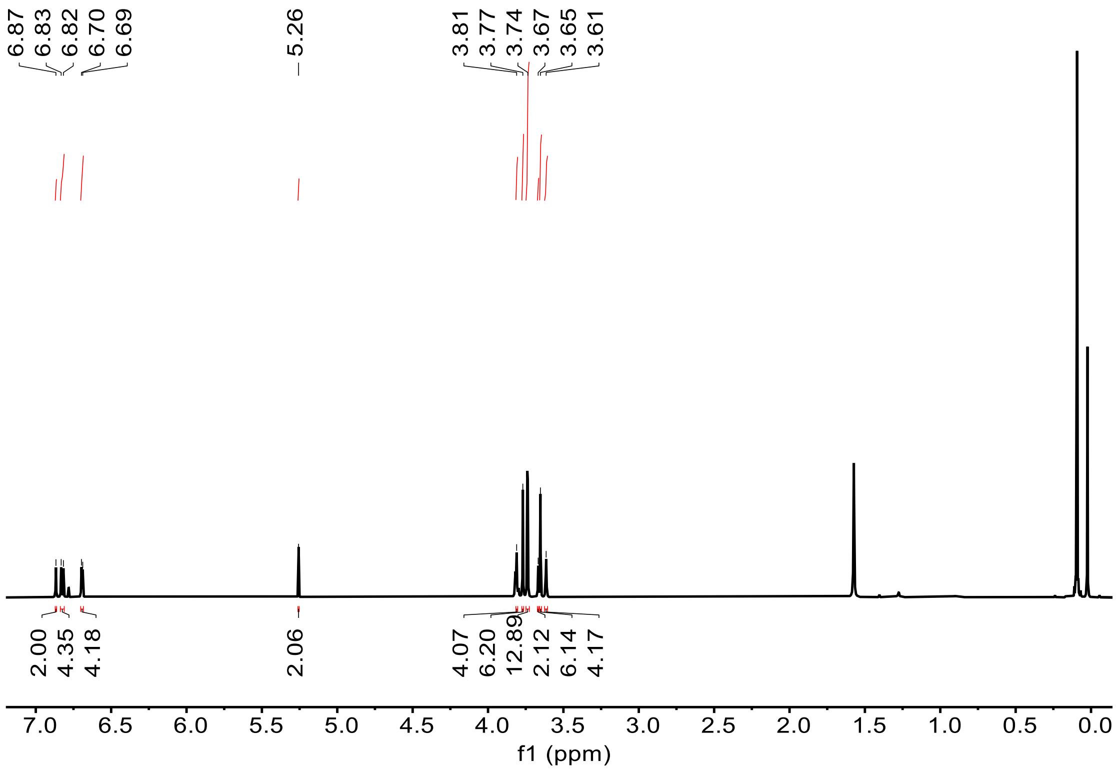

Figure S3. The ^{1}H NMR spectrum (400 MHz, $CDCl_3$, room temperature) of compound **P5-Q**.

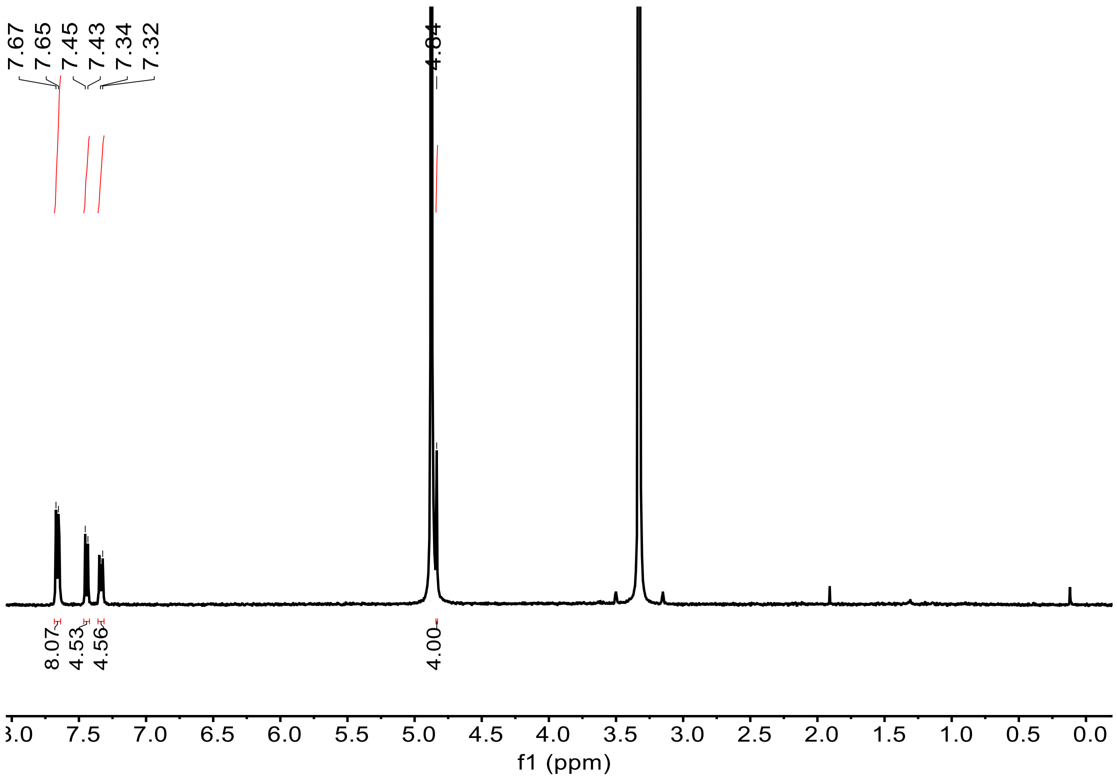

Figure S4. The ¹H NMR spectrum (400 MHz, d_4-Methanol, room temperature) of compound **M1**.

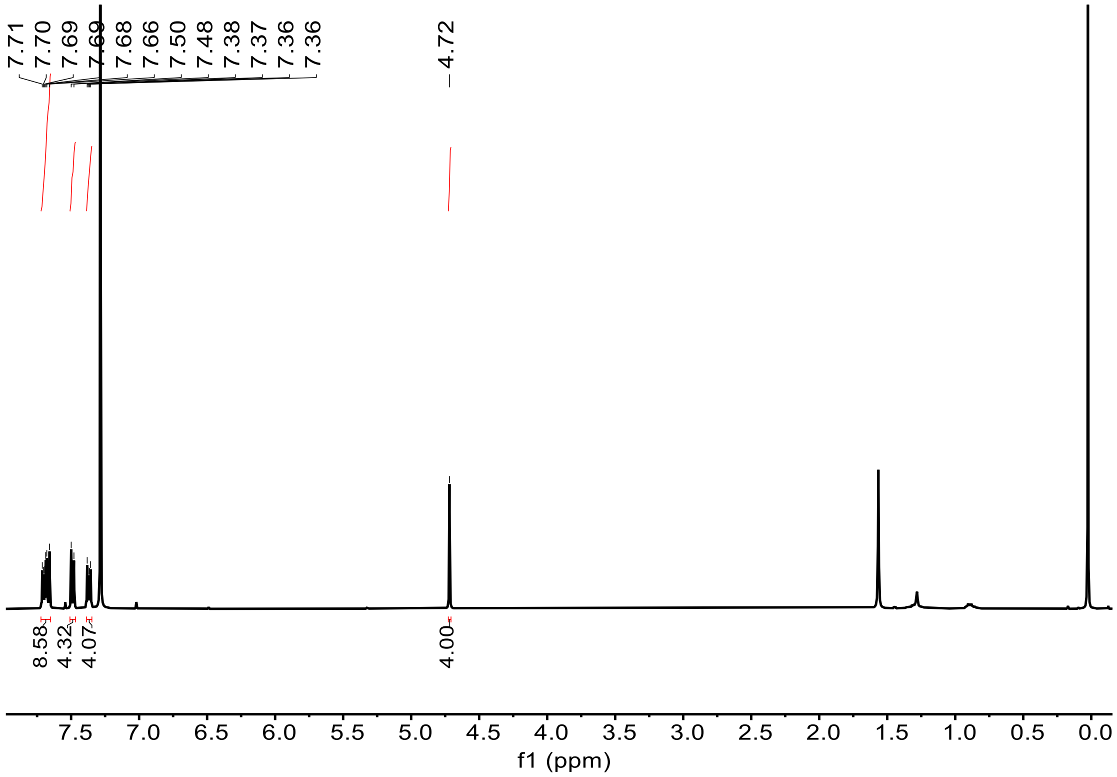

Figure S5. The ¹H NMR spectrum (400 MHz, $CDCl_3$, room temperature) of compound **M2**.

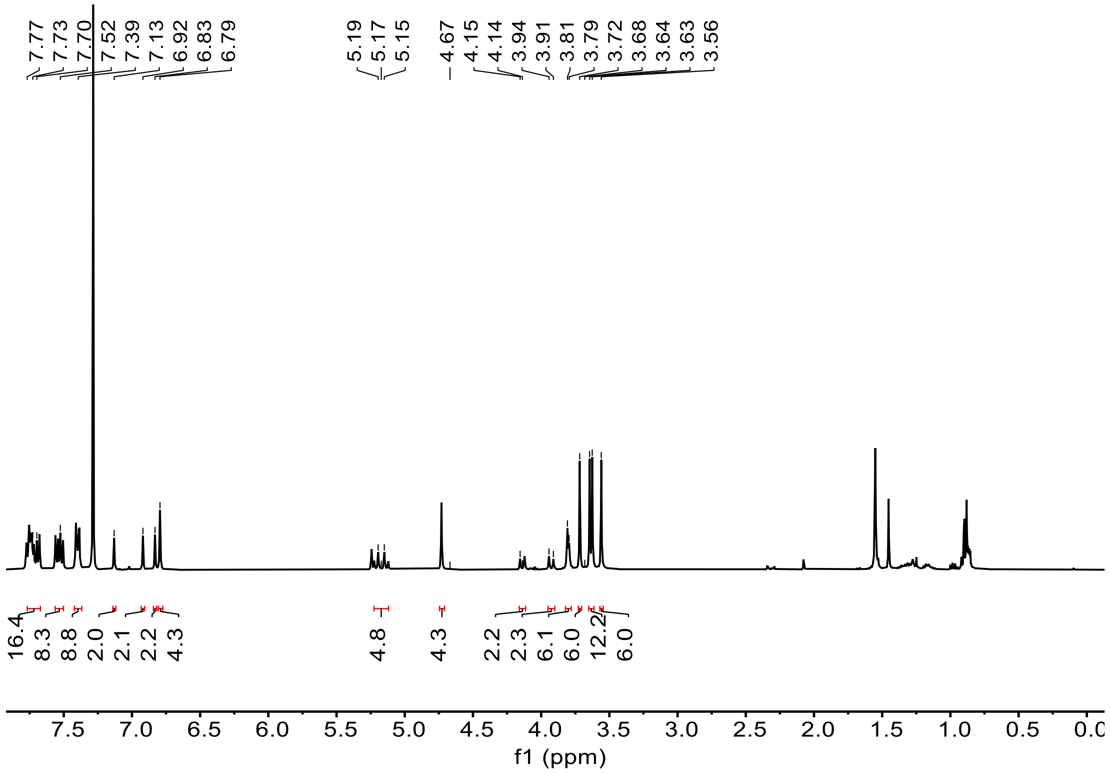

Figure S6. The ^{1}H NMR spectrum (400 MHz, $CDCl_3$, room temperature) of compound **A-Br**.

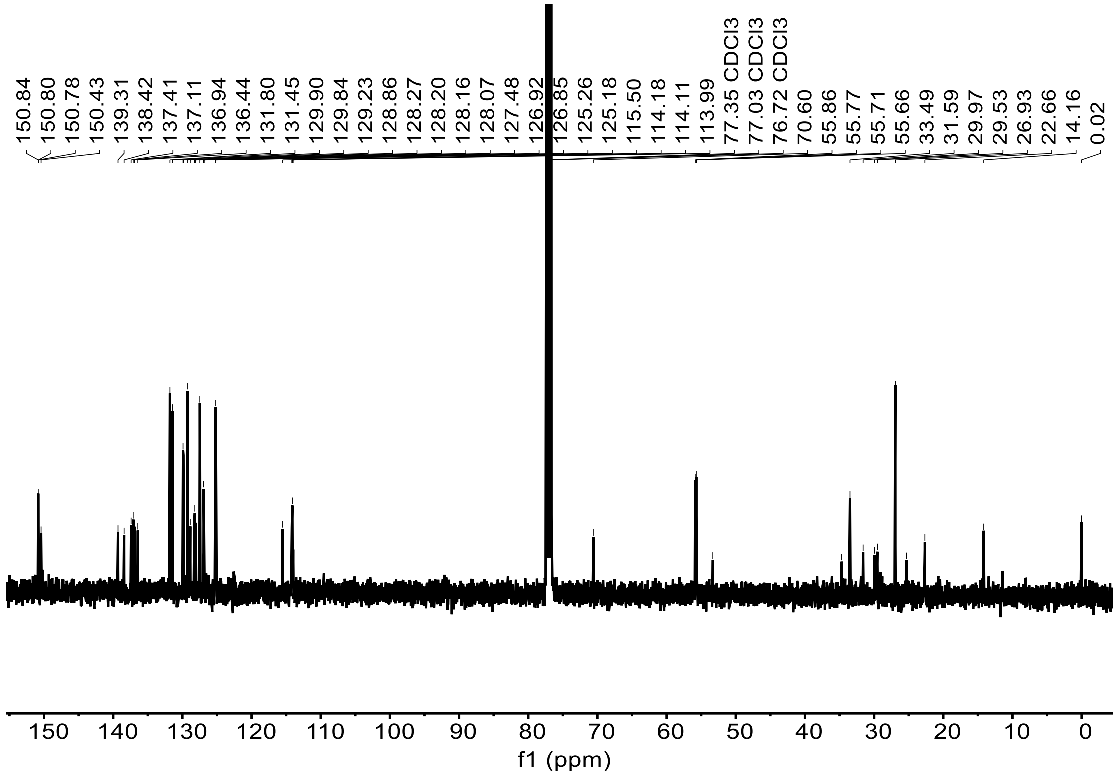

Figure S7. The ^{13}CNMR spectrum (400 MHz, $CDCl_3$, room temperature) of compound **A-Br**.

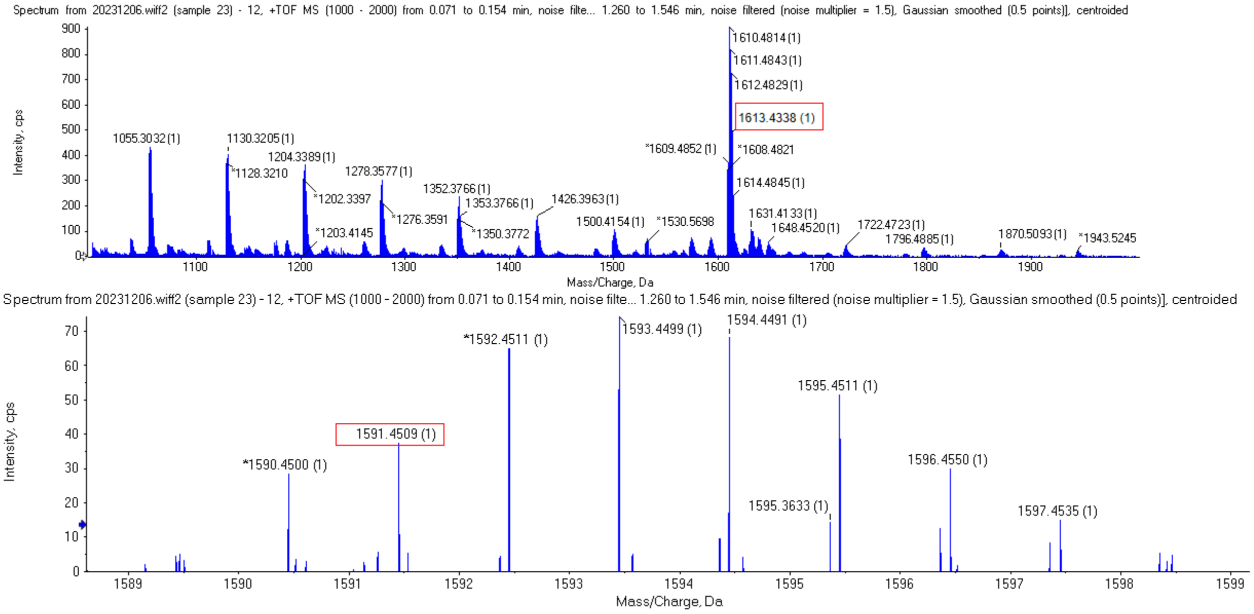

Figure S8. The Electrospray ionization mass spectrum of **A-Br**.

calcd ($C_{99}H_{85}Br_2O_{10}$]$^+$), *m/z* = 1591.4509, found: *m/z* = 1591.4509

calcd ([$C_{99}H_{84}Br_2O_{10}Na$]$^+$), *m/z* = 1613.4329, found: *m/z* = 1613.4338

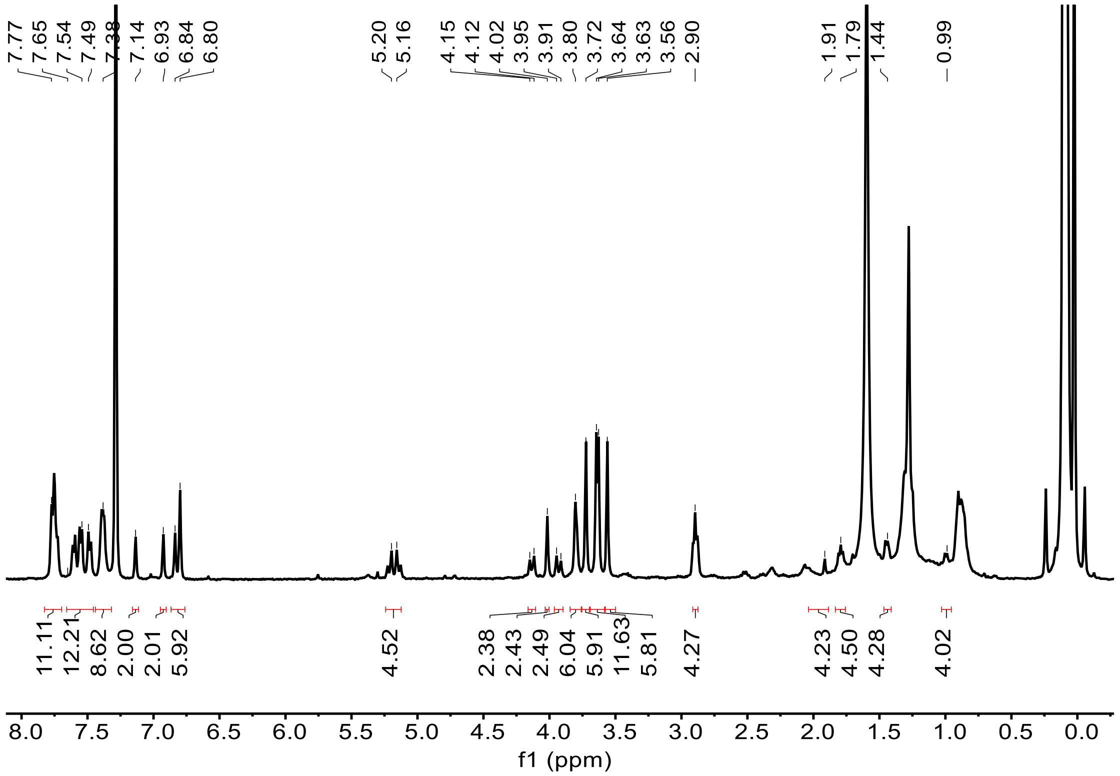

Figure S9. The ^{1}H NMR spectrum (400 MHz, $CDCl_3$, room temperature) of compound **A-NH$_2$**.

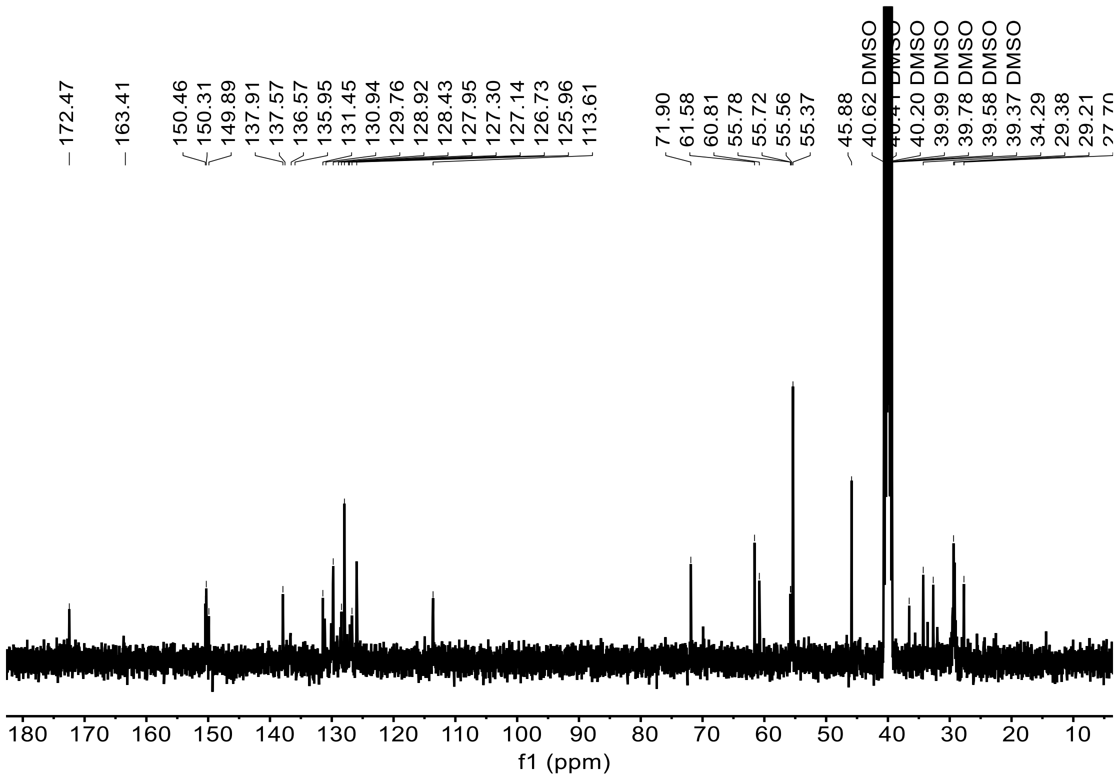

Figure S10. The ^{13}CNMR spectrum (400 MHz, DMSO, room temperature) of compound **A-NH$_2$**.

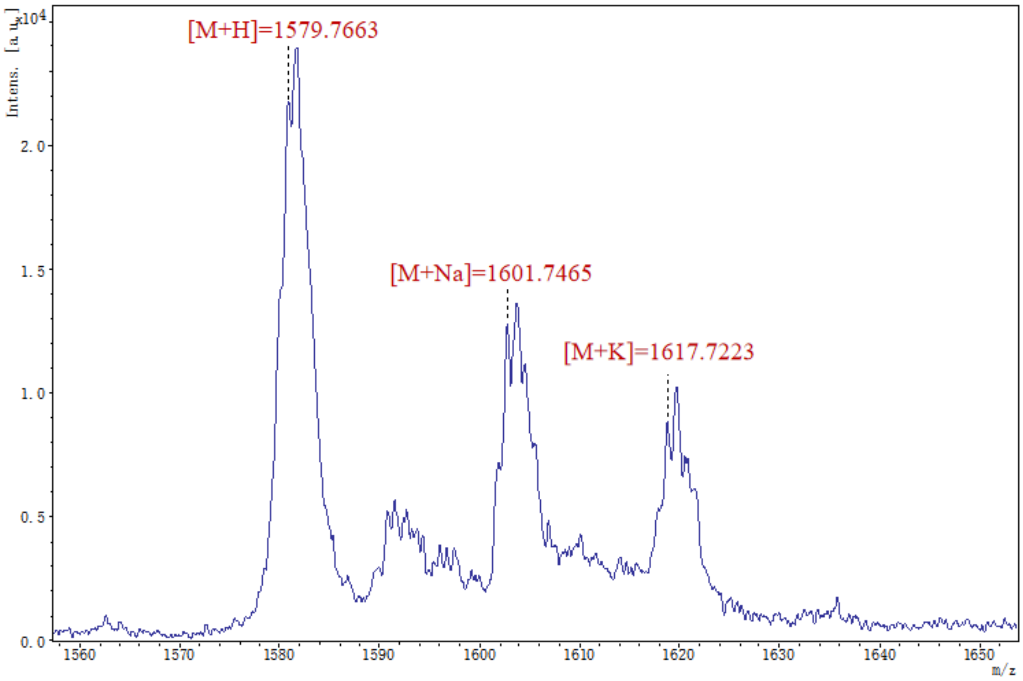

Figure S11. MALDI-TOF-HRMS spectra of **A-NH$_2$**.

calcd ([$C_{105}H_{102}N_4O_{10}Na$] $^+$), *m/z* = 1601.7494, found: *m/z* = 1601.7465

calcd ([$C_{105}H_{102}N_4O_{10}K$] $^+$), *m/z* = 1617.7233, found: *m/z* = 1617.7223

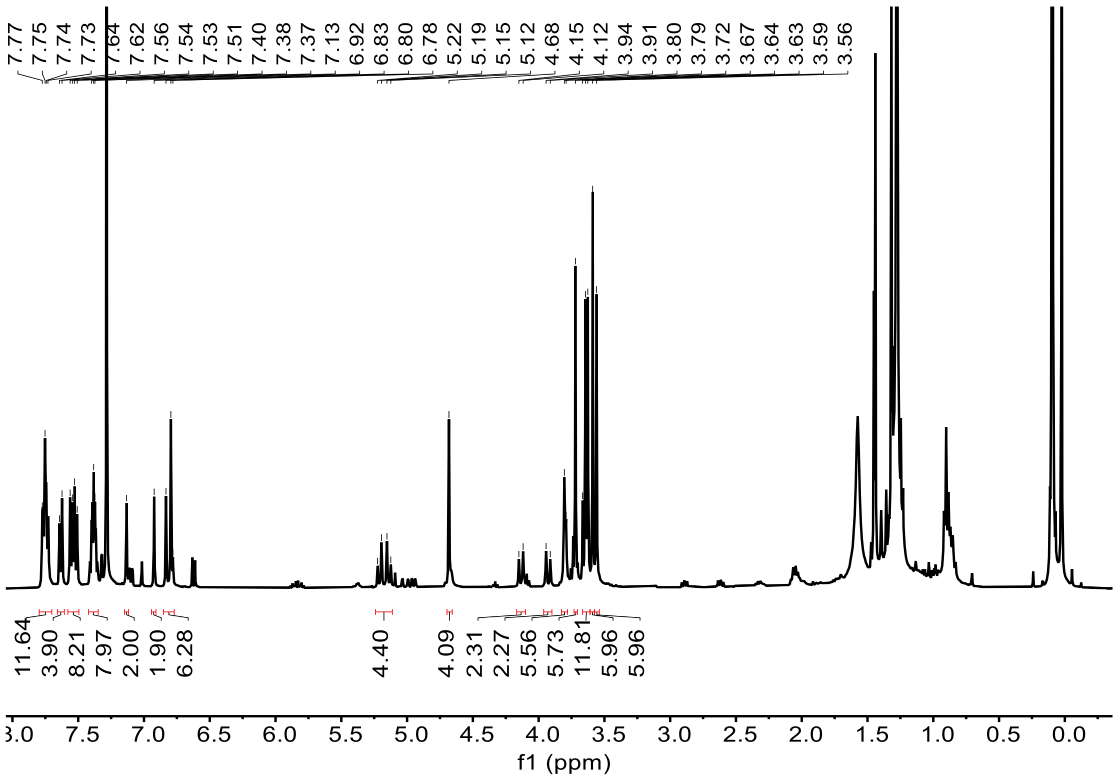

Figure S12. The ^{1}H NMR spectrum (400 MHz, $CDCl_3$, room temperature) of compound **A-1**.

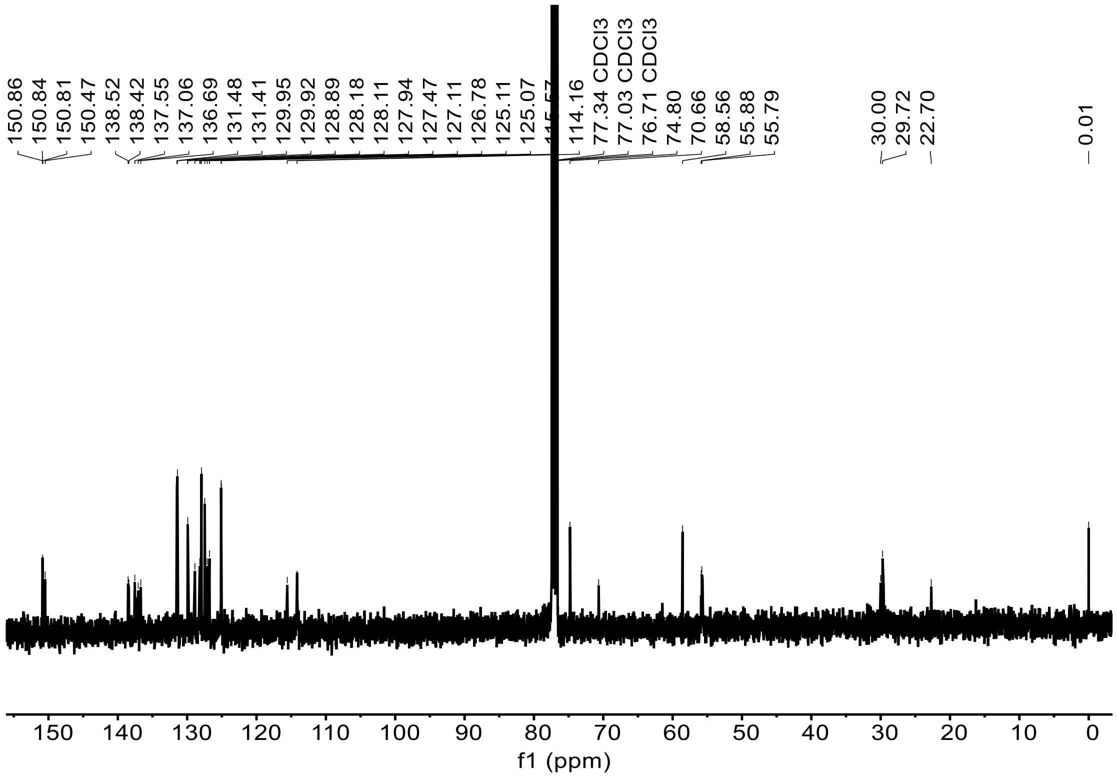

Figure S13. The ^{13}CNMR spectrum (400 MHz, $CDCl_3$, room temperature) of compound **A-1**.

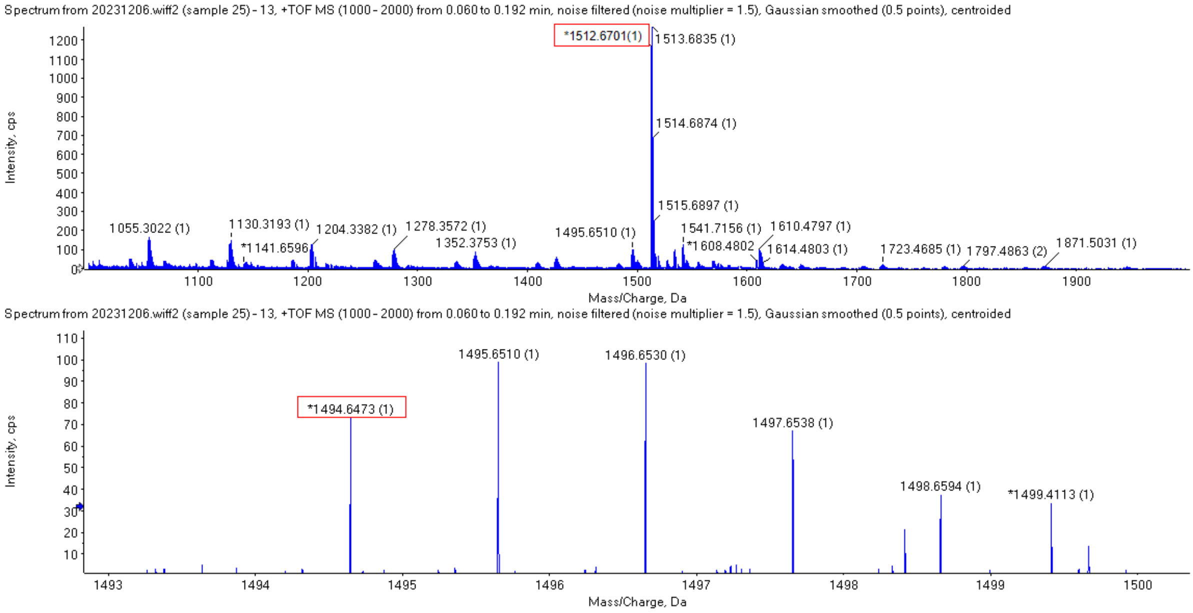

Figure S14. The Electrospray ionization mass spectrum of **A-1**.

calcd ($C_{101}H_{90}O_{12}$] $^{+}$), *m/z* = 1494.6432, found: *m/z* = 1494.6473

calcd ([$C_{101}H_{90}O_{12}NH_4$] $^{+}$), *m/z* = 1512.6771, found: *m/z* = 1512.6701

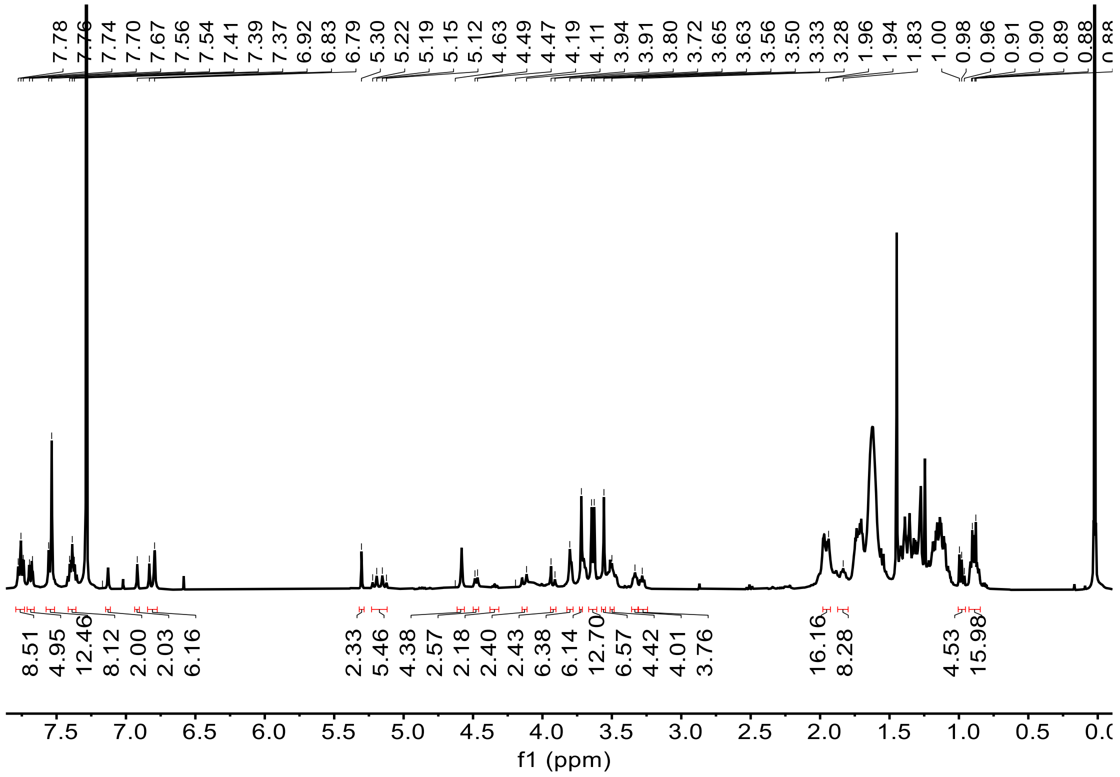

Figure S15. The ^{1}H NMR spectrum (400 MHz, $CDCl_3$, room temperature) of compound **A-2**.

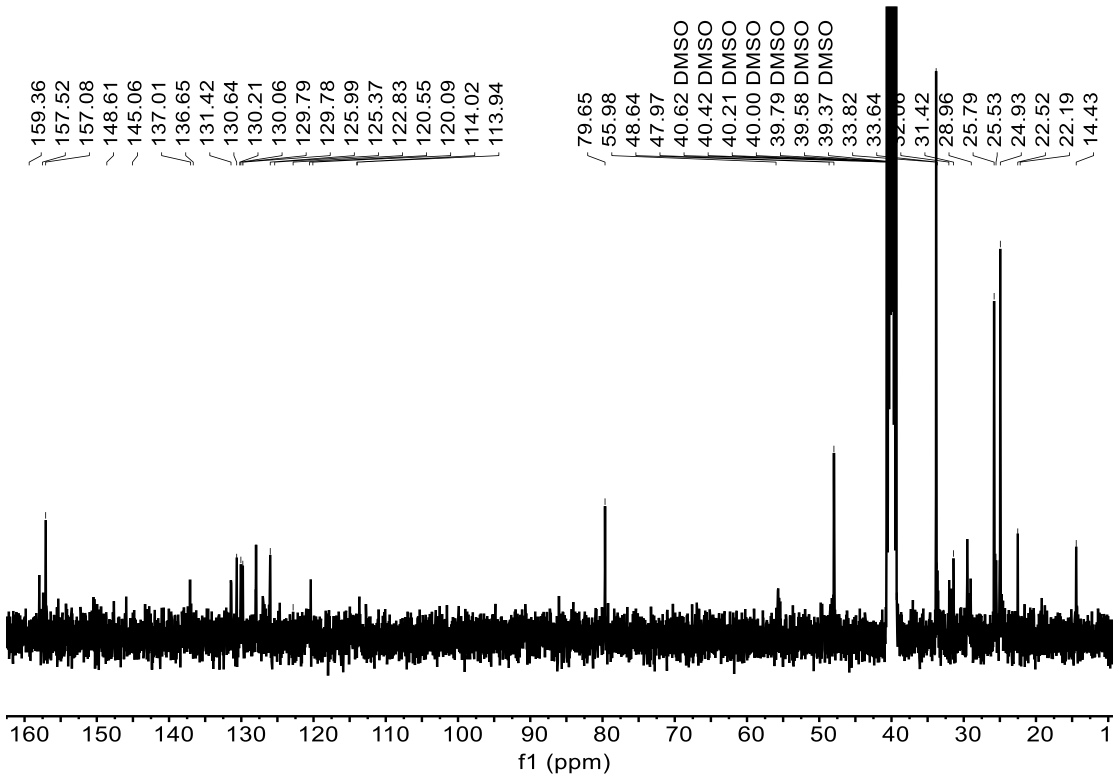

Figure S16. The ^{13}CNMR spectrum (400 MHz, DMSO, room temperature) of compound **A-2**.

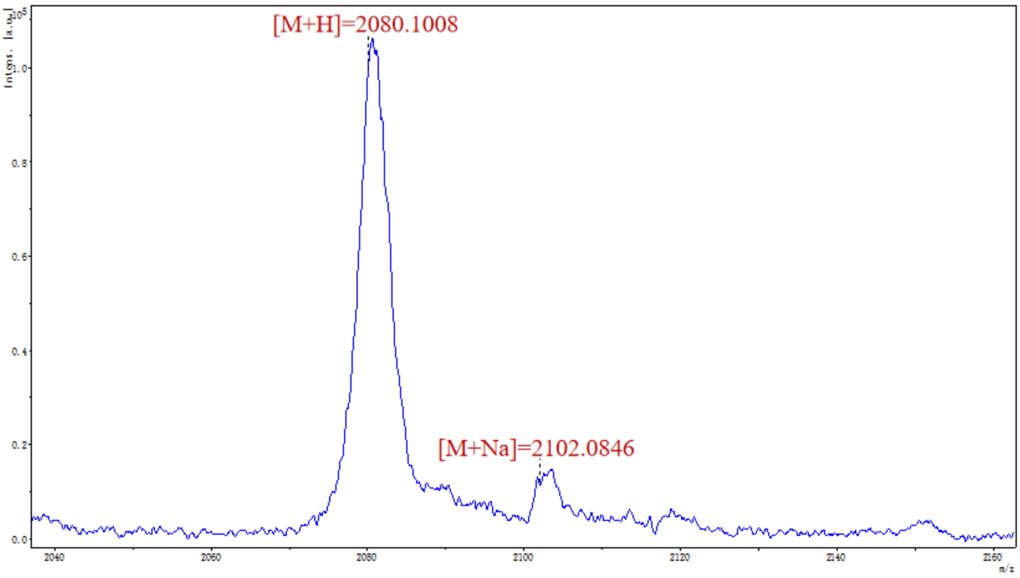

Figure S17. MALDI-TOF-HRMS spectra of **A-2**.

calcd ($[C_{133}H_{147}N_8O_{14}]^+$), *m/z* = 2080.1037, found: *m/z* = 2080.1008

calcd ($[C_{133}H_{147}N_8O_{14}Na]^+$), *m/z* = 2102.0856, found: *m/z* = 2102.0846

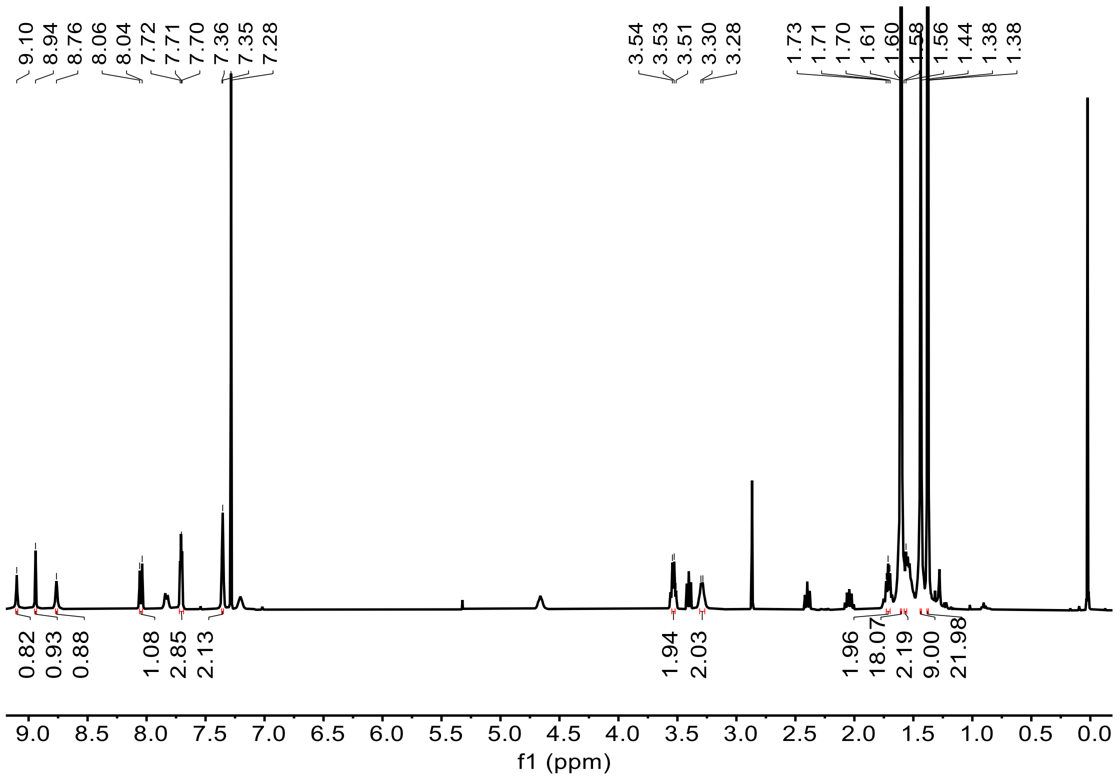

Figure S18. The ^{1}H NMR spectrum (400 MHz, $CDCl_3$, room temperature) of compound **S-0**.

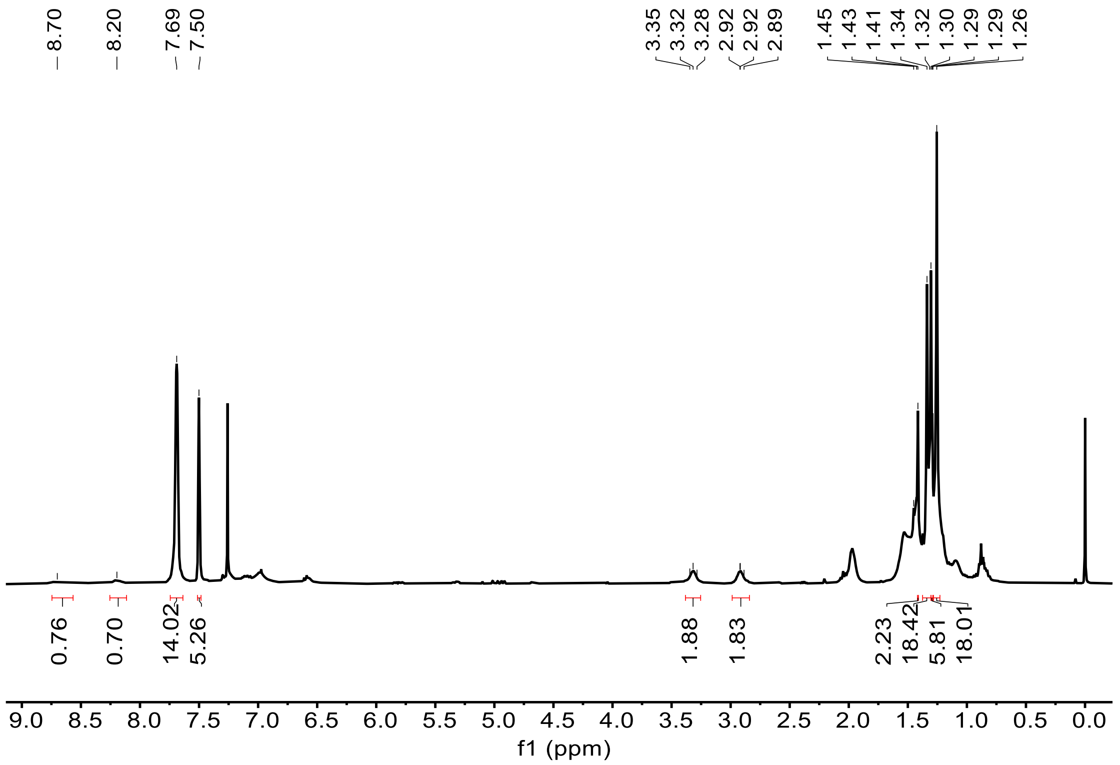

Figure S19. The ^{1}H NMR spectrum (400 MHz, $CDCl_3$, room temperature) of compound **S-2**.

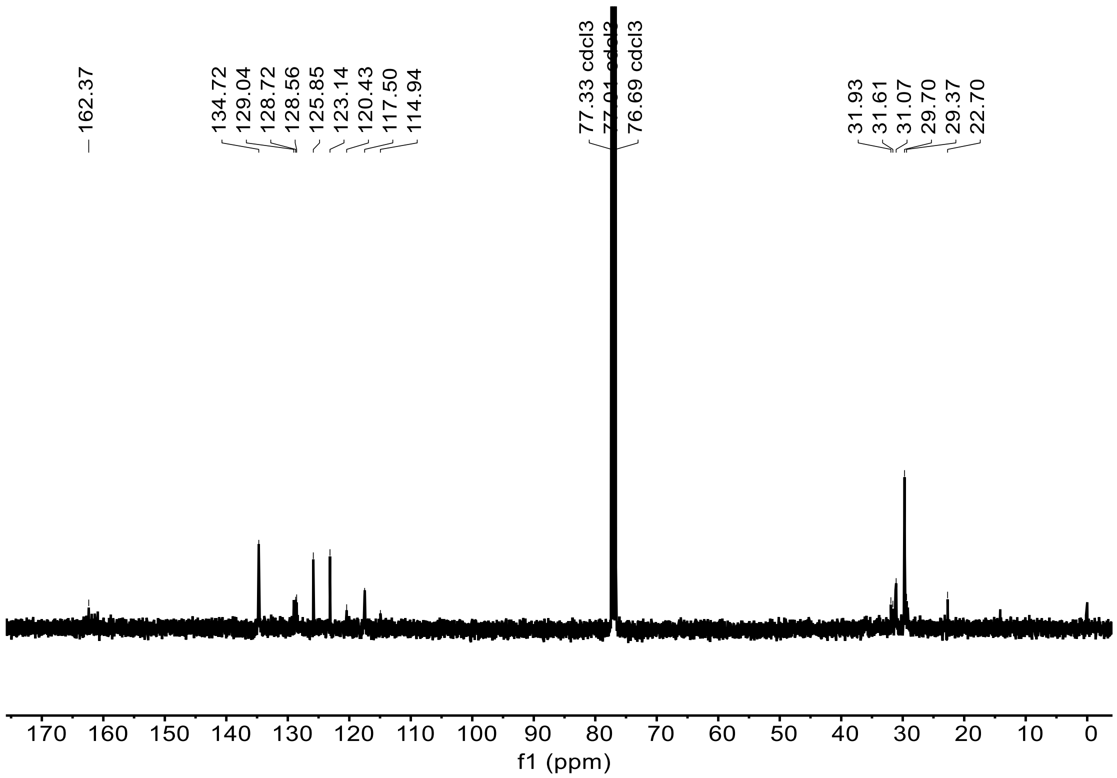

Figure S20. The ^{13}C NMR spectrum (101 MHz, $CDCl_3$, room temperature) of compound **S-2**.

S4. Photophysical details

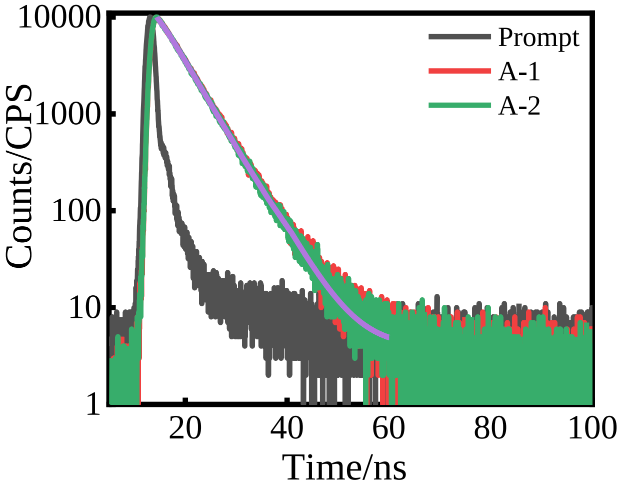

Figure S21. Fluorescence decay curves of **A-1**, **A-2** (10 μM) in Toluene, excited with nanosecond LED at 390 nm, 25°C.

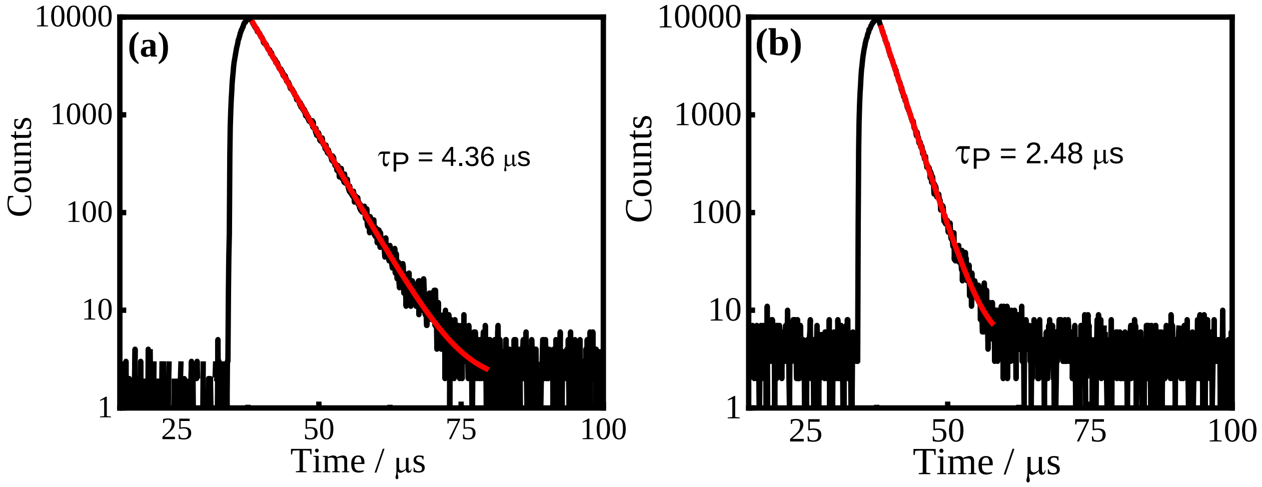

Figure S22. The phosphorescence lifetime decay curve of the photosensitizer molecule **S-1(a)**, **S-2(b)** in deoxygenated chloroform solution, λ_{ex} = 535 nm (Spectra LED), with the detection wavelength at the maximum phosphor emission wavelength.

S5. Host-guest binding details

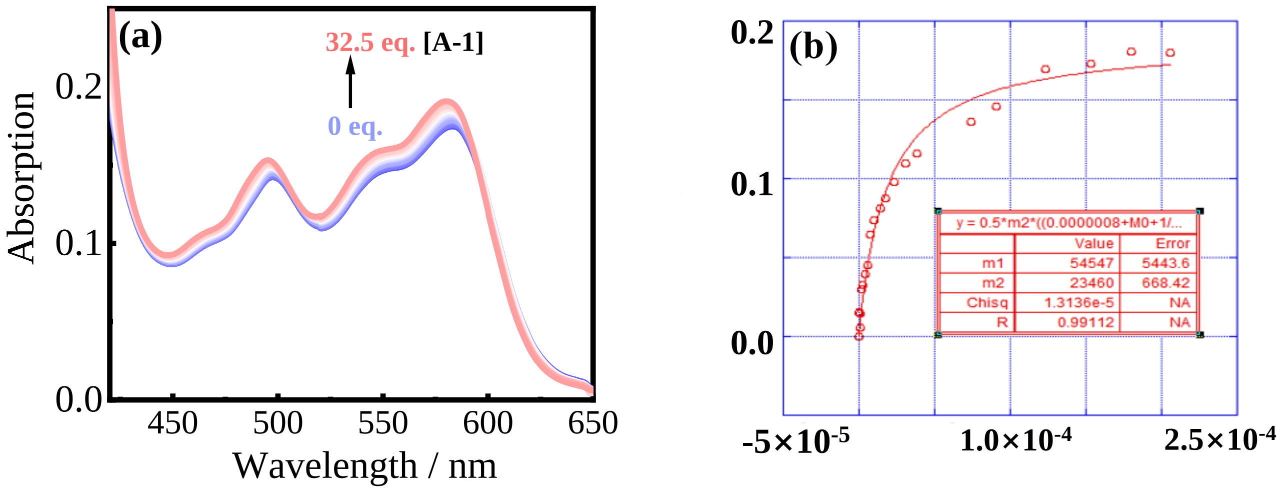

Figure S23. (a) UV-vis absorption spectra of the complexes **S-2⊂A-1** with different molar rations in $CHCl_3$ at 25°C, [**S-2**] = 20 μM, [**A-1**] = 0 mM-0.65 mM. (b) The non-linear curve-fitting (Uv-vis titrations) for the complexation of **A-1** with **S-2** (20 μM) in $CHCl_3$ at 25°C, the association constant (Ka) was 5.40 (±0.60) × 10^4 M^{-1}.

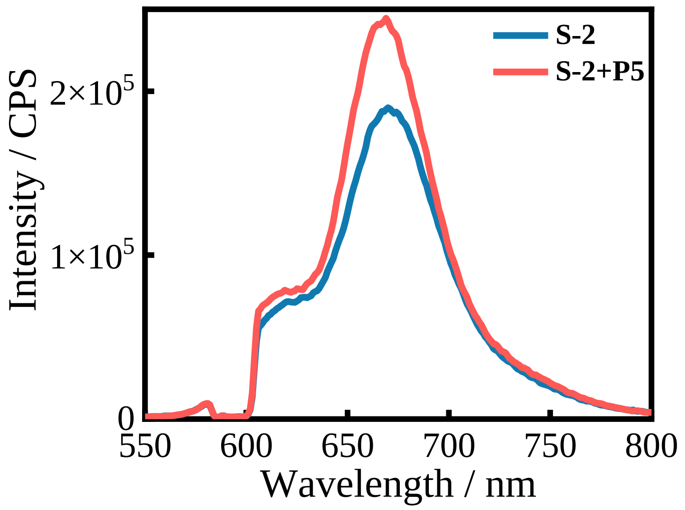

Figure S24. Emission photography of **S-2**, **S-2 + P5** measured in Toluene, excited by a CW laser (589 nm, 5.2 mW, slit = 0.6 nm) at 25°C, [**S-2**] = 5 μM, [**P5**] = 25 μM.

S6. Upconversion details

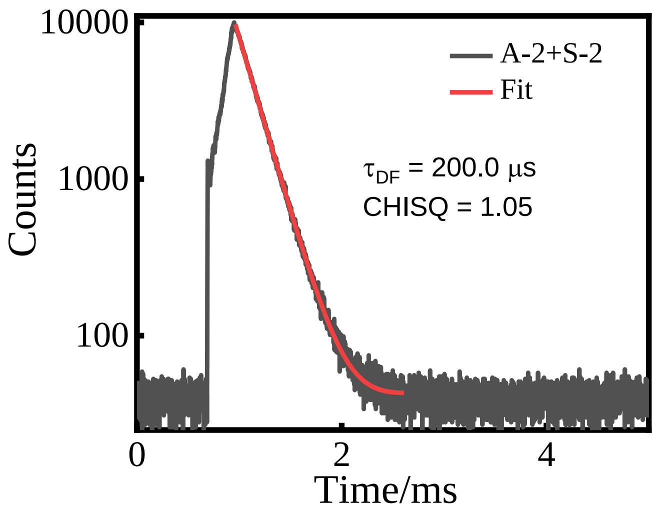

Figure S25. Delayed fluorescence observed the TTA upconversion with compound **S-2** as the triplet photosensitizer and **A-2** as the triplet acceptor of the upconverted fluorescence of **A-2** using **S-2** as the photosensitizer, λex = 589 nm, [**S-2**] = 5 μM; [**A-2**] = 25 μM; In deaerated Toluene, 25 °C.

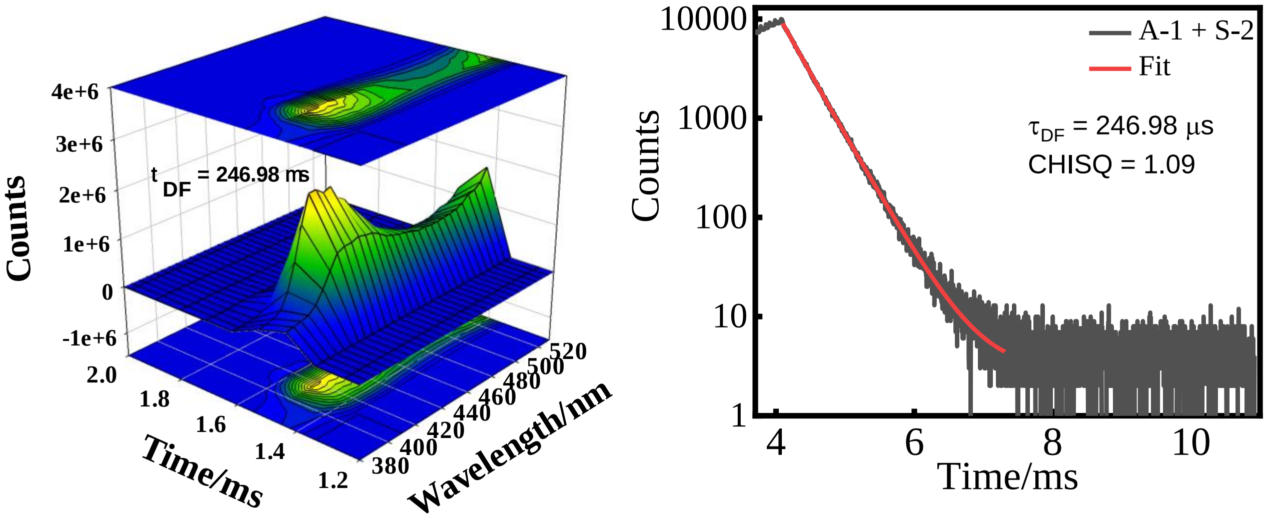

Figure S26. Time-resolved emission spectra (TRES) and delayed fluorescence observed the TTA upconversion of the upconverted fluorescence of **A-1** using **S-2** as the photosensitizer, λ_{ex} = 589 nm, [**S-2**] = 5 μM; [**A-1**] = 25 μM; In deaerated Toluene, 25 °C.

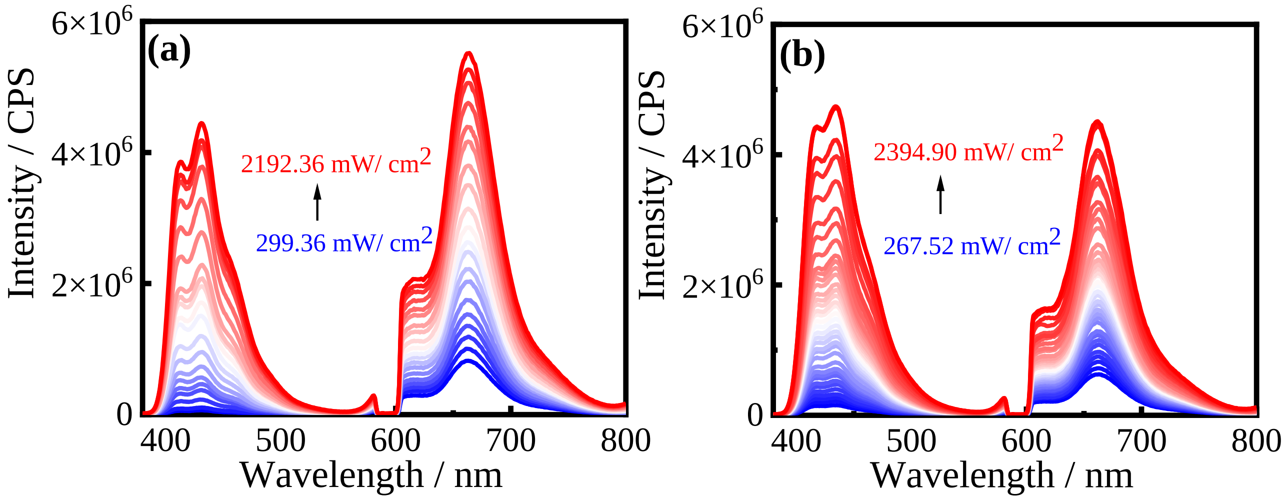

Figure S27. (a) The Excitation power dependency of the upconverted **A-1** emission with **S-2** as the sensitizer, the minimal and the maximal excitation power densities are 299.36 mW/ cm^2 and 2192.36 mW/ cm^2, respectively; (b) The Excitation power dependency of the upconverted **A-2** emission with **S-2** as the sensitizer , the minimal and the maximal excitation power densities are 267.52 mW/ cm^2 and 2394.90 mW/ cm^2, respectively; λ_{ex} = 589 nm, in deaerated Toluene, 25 °C. [**S-2**] = 5 μM, [**A-1**] = 25 μM, [**A-2**] = 25 μM.

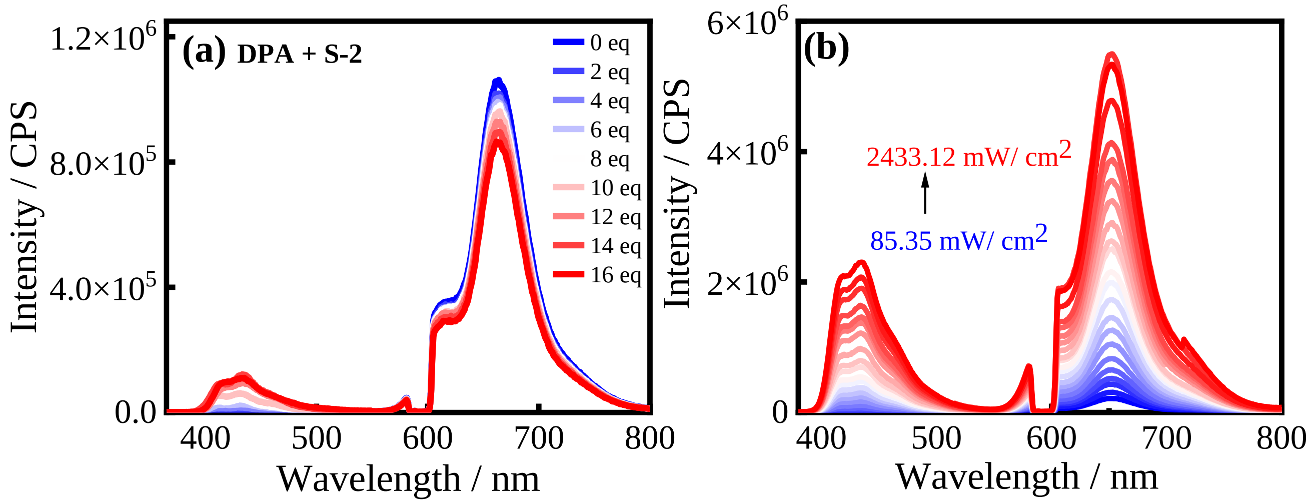

Figure S28. (a) UC emission spectra with **S-2** as the triplet photosensitizer and **DPA** as the acceptor with increasing concentration of **DPA**. The concentration of A-1 was 10 uM-80 uM, Excited with a 589nm laser (5.2 mW, slit= 0.6nm); (b) The Excitation power dependency of the upconverted **DPA** emission with **S-2** as the sensitizer, the minimal and the maximal excitation power densities are 85.35 mW/ cm^2 and 2433.12 mW/ cm^2, respectively; λ_{ex} = 589 nm, in deaerated Toluene, 25 °C. [**S-2**] = 5 μM, [**DPA**] = 25 μM.

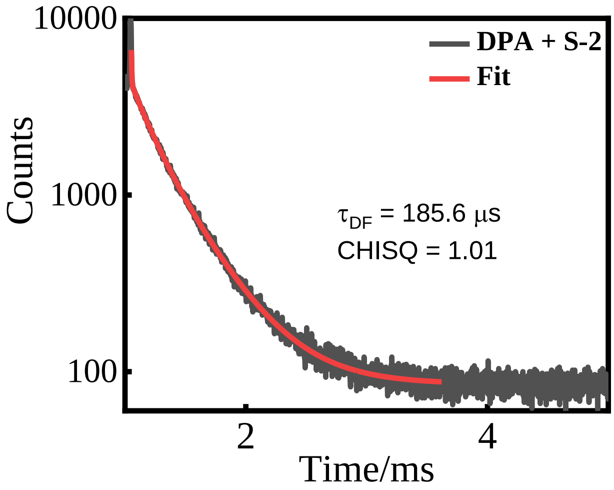

Figure S29. Delayed fluorescence observed the TTA upconversion with compound **S-2** as the triplet photosensitizer and **DPA** as the triplet acceptor, [**S-2**] = 5 μM, [**DPA**] = 50 μM, in deaerated Toluene, 25 °C.

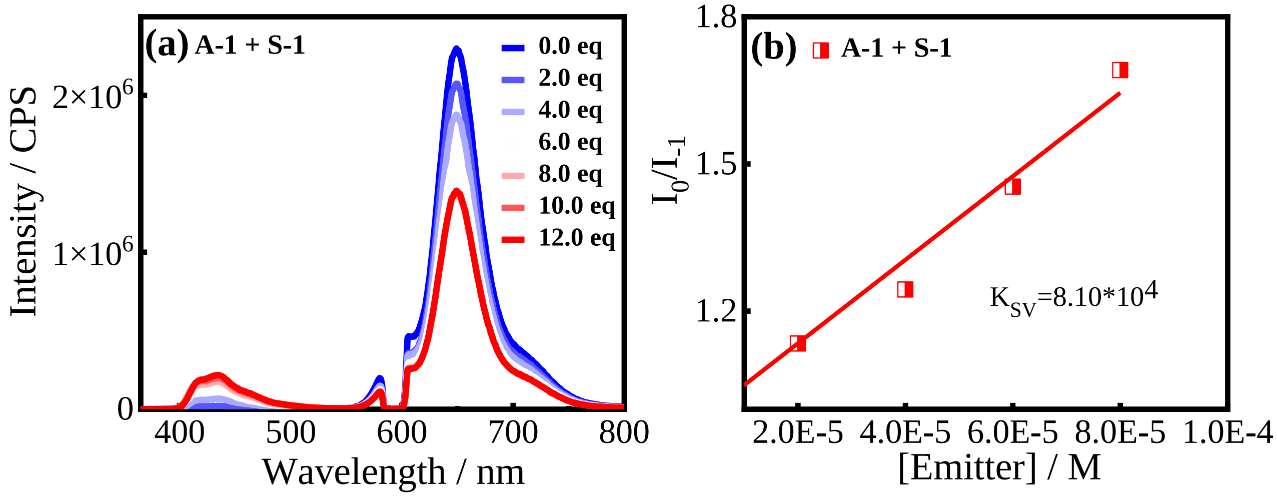

Figure S30. (a) UC emission spectra with **S-1** as the triplet photosensitizer and **A-1** as the acceptor with increasing concentration of **A-1**. The concentration of **A-1** was 10 µM - 60 µM, Excited with a 589nm laser (5.2 mW, slit= 0.6nm), in deaerated Toluene at 25°C; **(b)** Stern-Volmer Plots of I_0/I versus the concentration of annihilators; [**S-1**] = 5 µM.

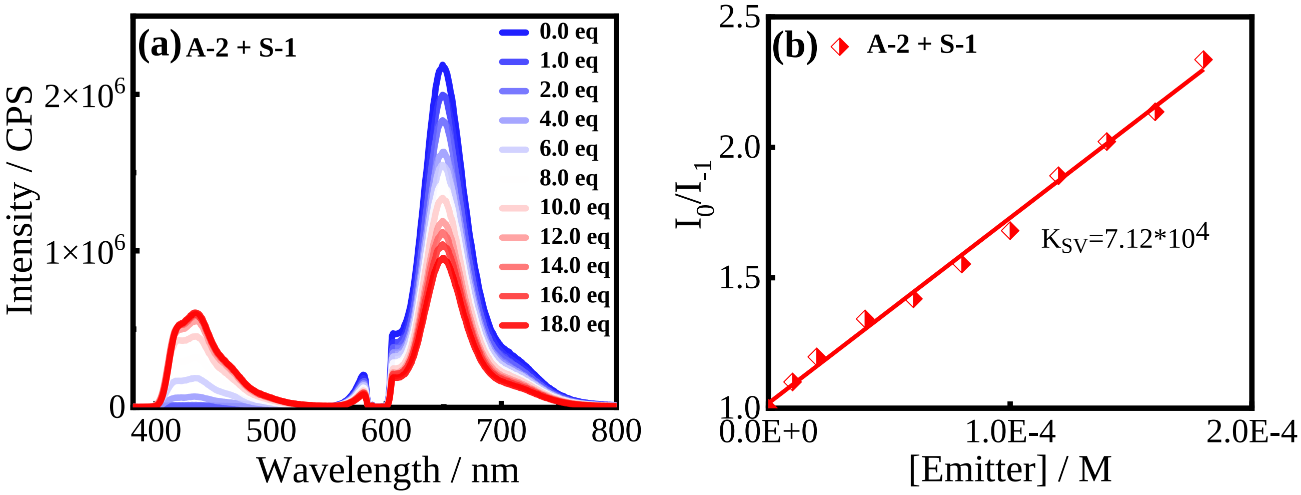

Figure S31. (a) UC emission spectra with **S-1** as the triplet photosensitizer and **A-2** as the acceptor with increasing concentration of **A-2**. The concentration of **A-2** was 10 µM - 90 µM, Excited with a 589nm laser (5.2 mW, slit= 0.6nm), in deaerated Toluene at 25°C; **(b)** Stern-Volmer Plots of I_0/I versus the concentration of annihilators; [**S-1**] = 5 µM.

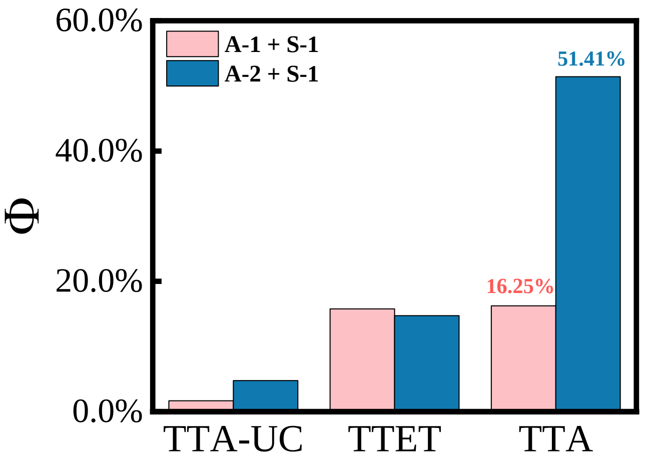

Figure S32. Histogram of Φ_{UC}, Φ_{TTET} and Φ_{TTA} of the UC systems with **S-1** as the sensitizer and **A-1**, **A-2**, **DPA** as the annihilator. [**S-2**] = *5* mM, [**A-1**] = *25*mM, [**A-2**] = *25* mM and [**DPA**] = *50* mM in toluene.

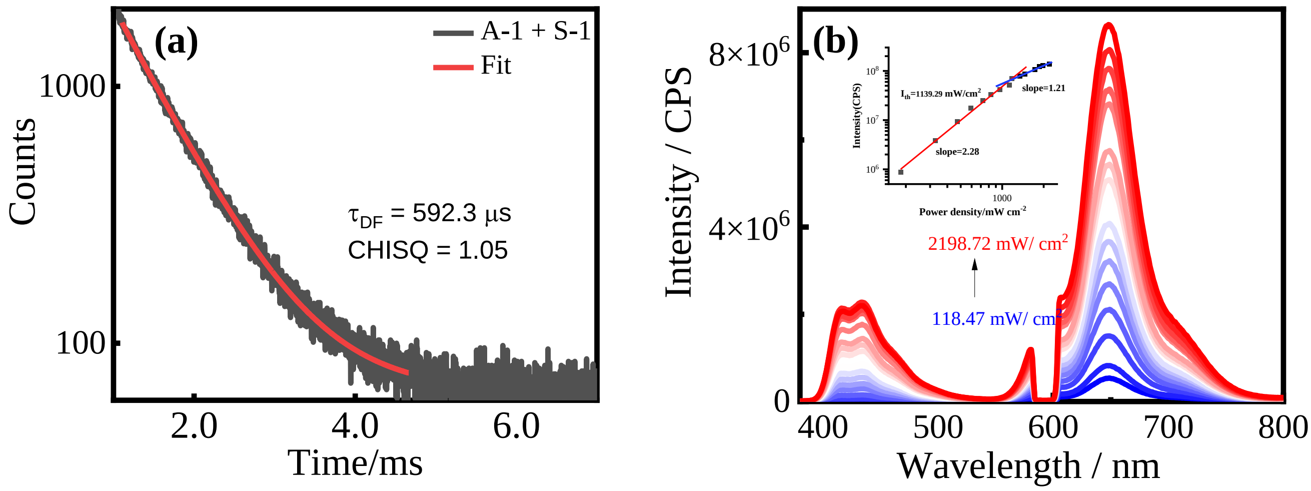

Figure S33. (a) Delayed fluorescence observed the TTA upconversion of the upconverted fluorescence of **A-1** using **S-1** as the photosensitizer; (b) The Excitation power dependency of the upconverted **A-1** emission with **S-1** as the sensitizer (Insert: the normalized integrated emission intensity plotted as a function of normalized incident light power), the minimal and the maximal excitation power densities are 118.47 mW/ cm^2 and 2198.72 mW/ cm^2, respectively; λ_{ex} = 589 nm, in deaerated Toluene, 25 °C. [**S-1**] = 5 μM, [**A-1**] = 25 μM.

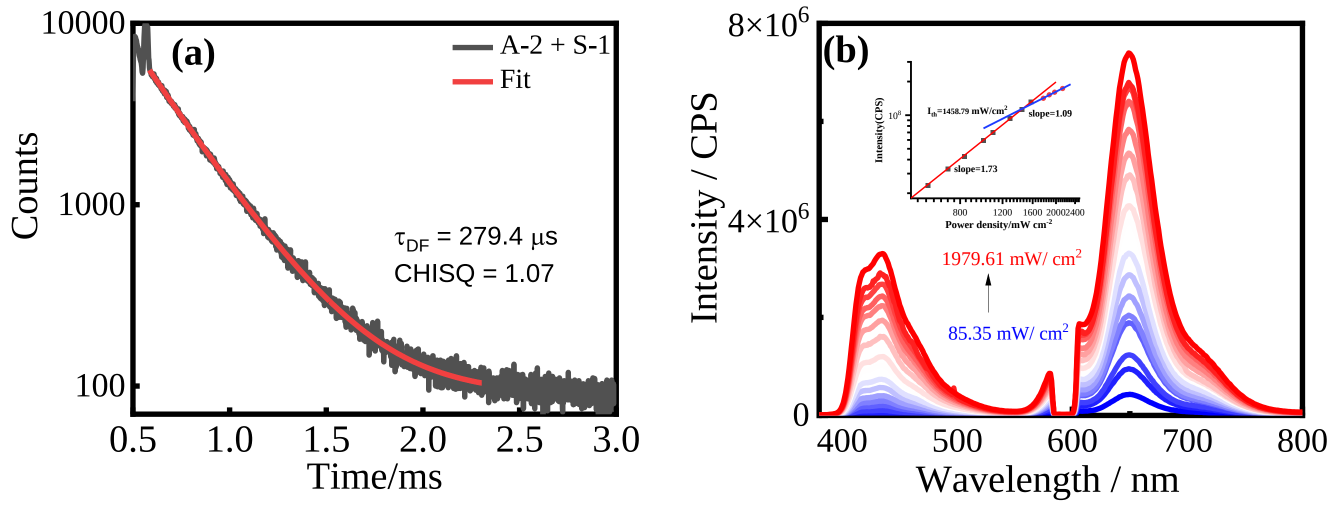

Figure S34. (a) Delayed fluorescence observed the TTA upconversion of the upconverted fluorescence of **A-2** using **S-1** as the photosensitizer; (b) The Excitation power dependency of the upconverted **A-2** emission with **S-1** as the sensitizer (Insert: the normalized integrated emission intensity plotted as a function of normalized incident light power), the minimal and the maximal excitation power densities are 85.35 mW/ cm^2 and 1979.61 mW/ cm^2, respectively; λ_{ex} = 589 nm, in deaerated Toluene, 25 °C. [**S-1**] = 5 μM, [**A-2**] = 25 μM.

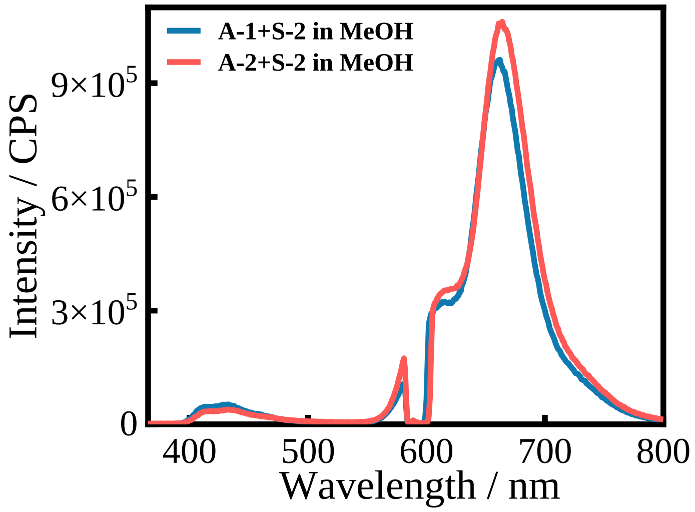

Figure S35. UC emission spectra with **S-2** as the triplet photosensitizer and **A-1**, **A-2** as the acceptor, in deaerated MeOH, [**S-2**] =5 μM, [**A-1**] = 25 μM, [**A-2**] = 25 μM.
